# Thermodynamics Constrains Allometric Scaling of Optimal Development Time in Insects

**DOI:** 10.1371/journal.pone.0084308

**Published:** 2013-12-31

**Authors:** Michael E. Dillon, Melanie R. Frazier

**Affiliations:** 1 Department of Zoology & Physiology and Program in Ecology, University of Wyoming, Laramie, Wyoming, United States of America; 2 Pacific Coastal Ecology Branch, Western Ecology Division, United States Environmental Protection Agency, Newport, Oregon, United States of America; Michigan State University, United States of America

## Abstract

Development time is a critical life-history trait that has profound effects on organism fitness and on population growth rates. For ectotherms, development time is strongly influenced by temperature and is predicted to scale with body mass to the quarter power based on 1) the ontogenetic growth model of the metabolic theory of ecology which describes a bioenergetic balance between tissue maintenance and growth given the scaling relationship between metabolism and body size, and 2) numerous studies, primarily of vertebrate endotherms, that largely support this prediction. However, few studies have investigated the allometry of development time among invertebrates, including insects. Abundant data on development of diverse insects provides an ideal opportunity to better understand the scaling of development time in this ecologically and economically important group. Insects develop more quickly at warmer temperatures until reaching a minimum development time at some optimal temperature, after which development slows. We evaluated the allometry of insect development time by compiling estimates of minimum development time and optimal developmental temperature for 361 insect species from 16 orders with body mass varying over nearly 6 orders of magnitude. Allometric scaling exponents varied with the statistical approach: standardized major axis regression supported the predicted quarter-power scaling relationship, but ordinary and phylogenetic generalized least squares did not. Regardless of the statistical approach, body size alone explained less than 28% of the variation in development time. Models that also included optimal temperature explained over 50% of the variation in development time. Warm-adapted insects developed more quickly, regardless of body size, supporting the “hotter is better” hypothesis that posits that ectotherms have a limited ability to evolutionarily compensate for the depressing effects of low temperatures on rates of biological processes. The remaining unexplained variation in development time likely reflects additional ecological and evolutionary differences among insect species.

## Introduction

A rich literature investigates the relationship between body size and life history traits of diverse organisms [1]–[3]. In particular, development time has long fascinated ecologists because of its compounding effects on organism fitness and therefore maximum population growth rates (intrinsic rates of increase, *r*) [4], [5]. That development time should vary with body size is not controversial: it should take longer to build more tissue. However, controversy surrounds the precise nature of the relationship between body size and development time (the allometric scaling exponent and intercept) which has important implications at multiple hierarchical levels and has been measured for diverse organisms [2], [6]. The allometry of development time can reveal capacities and limits of the underlying processes of cell division and differentiation [7], as well as informing discussions of the life-history implications [2] and ecological consequences of body size [1]. For example, models addressing key questions about body size evolution such as why developing in colder temperatures typically results in larger body sizes for ectotherms (i.e., the temperature size rule; [8]), often hinge on the relative rates of growth (development time) and differentiation [9], [10]. Therefore, a better understanding of the scaling relationship between body size and development time can critically alter the conclusions of such models. At the ecological scale, differences in the scaling of development time with body size can dramatically alter predictions (based on body size) of organism population growth rates, space usage and resource demands [11], [12]. Finally, variation in development time not explained by body size begs explanation by other physiological and ecological hypotheses.

Documenting the empirical relationship between body size and life history traits is important in its own right, but there has also been a strong interest in mechanistic explanations for these patterns [11], [13], [14]. The metabolic theory of ecology (MTE) attempts to explain the scaling of biological variables with body mass using fundamental principles from physics and chemistry [11], [14]. According to the MTE, organisms supply their tissues via fractal-like branching networks which are space filling and optimized to minimize transportation costs [14]. Given these characteristics, metabolic rate is predicted to scale with mass^0.75^ [14]. Furthermore, the MTE predicts that, although metabolic rates may vary among taxonomic groups (i.e. different intercepts), the slope of the scaling relationship should be consistent across taxa [14], regardless of phylogenetic effects [15]. Ultimately the relationship between body mass and metabolic rate has cascading effects, resulting in quarter-power scaling predictions at the individual, population, and ecological levels [11]. Although controversial [16], [17], the MTE is appealing because it provides a potentially powerful approach to linking the physiology of individual organisms to ecological processes simply by knowing body size and temperature [11].

The ontogenetic growth model (OGM) builds on the MTE to mechanistically describe organism development in terms of the bioenergetics of growth [18]. The OGM posits that organism growth rates depend on the balance between how energy consumed by organisms is devoted to new growth relative to maintenance of existing biomass. Assuming that whole organism metabolic rate scales with mass^0.75^ and that the energy required to create and maintain tissues does not vary with body size or tissue type, the OGM predicts that the development time of diverse organisms will scale with mass^0.25^ [12], [18]–[20].

Many empirical studies find quarter power scaling of growth rates (and times) with body size, confirming the prediction of the OGM. These studies, largely limited to birds and mammals, suggest that embyronic and post-embyonic growth times, though variable, scale with body mass to the roughly 0.25 power [1], [2], [21], [22]. Beyond these groups, maturation times from viruses to mammals were found to scale with adult mass to the 0.26 power [6].

Despite the controversy and the large body of literature on vertebrate homeotherms, there is a dearth of synthetic studies on the scaling of development time with body size in ectotherms, and particularly in insects. There has been some interest in the effects of egg or neonate mass on embryonic development time [23]–[25], but, to our knowledge, there are no synthetic studies addressing the scaling of egg to adult development time with adult body size. Development times of diverse insects have been measured for over a century [26], [27] due to their abundance in virtually all ecosystems, and to their far-reaching and profound ecological and economic impacts. Given the abundance of independent studies of their development times, insects provide an ideal opportunity to test whether the scaling of development time with body size is consistent with empirical fits in other taxa and with theoretical expectations from the OGM.

One of the challenges of studying the scaling of development time in insects and other ectothermic organisms is the strong effect of temperature, independent of body size, on rates of development [20], [28], [29]. For a given species, development time decreases with increasing temperature to a minimum value (“minimum or optimal development time”) occurring at some optimal temperature (*T*_opt_). At temperatures higher than *T*_opt_, development takes longer, likely due to the physiological challenges of dealing with stressfully hot temperatures [30]. Among diverse insects, *T*_opt_ for intrinsic rates of population growth, *r*, is correlated with environmental temperature [31], suggesting that minimum development time is an ecologically relevant metric of a species’ ability to mature in its native environment. Although insects have adapted to a wide range of thermal environments, they do not fully compensate for the depressing effects of low temperatures on rates of biological processes (the “warmer is better” hypothesis; [31], [32]). Consequently, species adapted to cold environments are predicted to have longer minimum development times than warm-adapted species, at their respective *T*_opt_.

Here we examine the scaling of minimum development time with body mass and *T*_opt_ in a large and diverse insect data set to determine whether: (1) minimum development time scales with body mass according to theoretical predictions [18], [19], (2) body size alone explains most of the variation in minimum development time, and (3) whether adaptation of insects to different thermal environments drives differences in minimum development time (a test of the “warmer is better” hypothesis) [32].

## Materials and Methods

**Figure 1:**
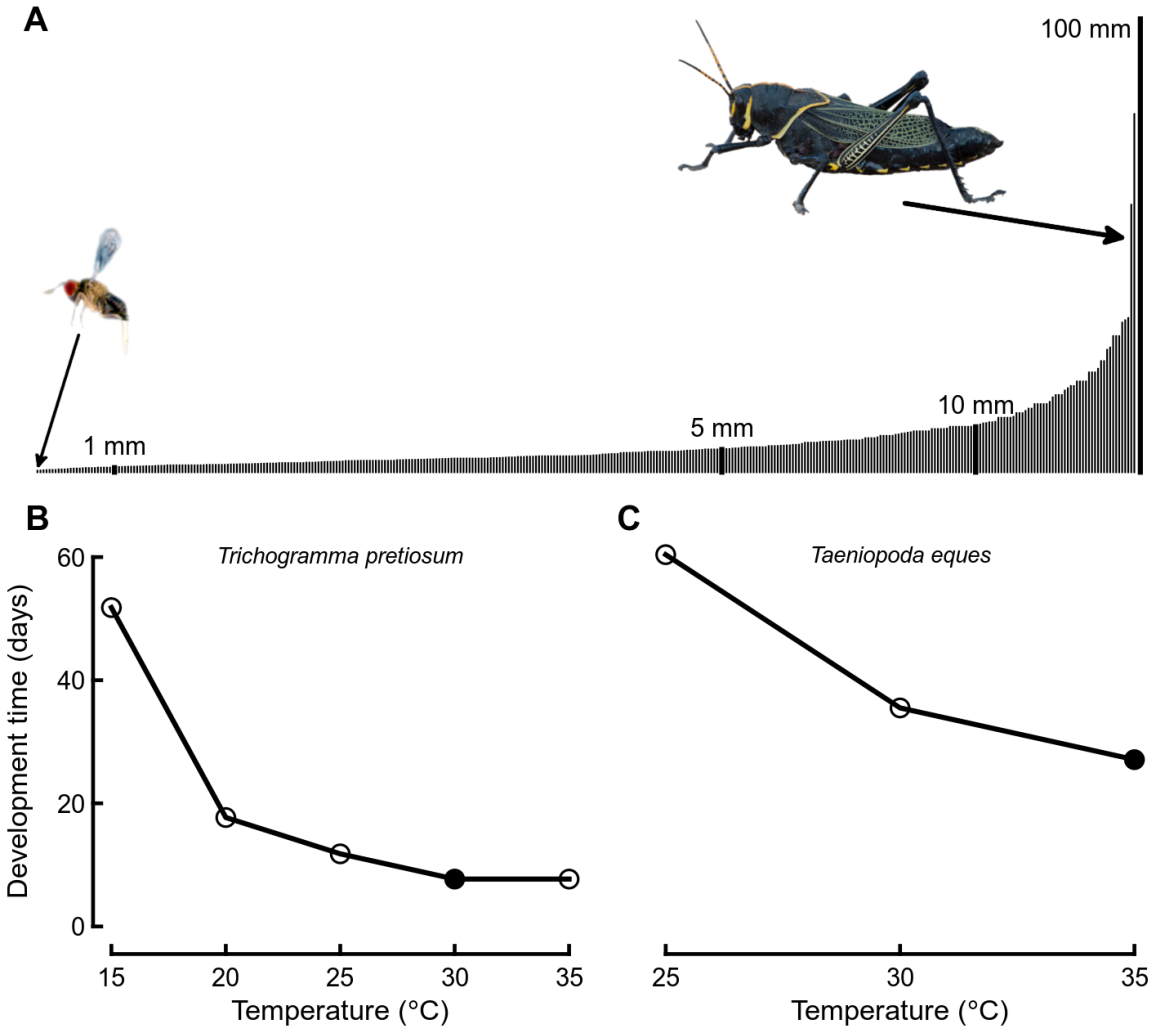
Insect body lengths and representative reaction norms for development time. (A) The length of each insect species included in the study (N = 361, Table S1) is represented by a vertical bar, arranged from smallest to largest, with vertical scale bars at 1, 5, 10, and 100 mm. The parasitic wasp (*Trichogramma pretiosum* ∼0.3 mm) is shown magnified 50× for comparison with the grasshopper (*Taeniopoda eques* ∼59 mm) which is shown to scale. Development time data for these two species are given in lower plots (B, C). For each species, development times were measured at a number of constant temperatures and the minimum development time and temperature at which that occurred (filled points) were used in subsequent analyses. (Wasp: P. Flinn, USDA, grasshopper: Wesley Sprinkle, CC).

We compiled literature measurements of egg to adult development time (days) for 361 insect species from 94 families and 16 orders (Table S1). For each species, development time was measured at 2–11 constant temperatures, with more than 80% of species measured at 4 or more temperatures (Table S1). In general, insect development time decreases with increasing temperature until it begins to level off or even increase at stressfully high temperatures (Fig. 1B, C) [12], [33], [34]. We determined the minimum development time (*T*_dev_) and the temperature at which that minimum occurred (*T*_opt_) for each species (Fig. 1B, C filled points). When multiple independent estimates of minimum development time were available for a single species, we used the minimum available estimate.

For each species, we determined mean body length (the most commonly reported measure of insect size) from the literature or by personal communication with authors or specialists. When a range of body lengths were available, we used the midpoint. Body lengths spanned nearly 2.5 orders of magnitude, from 0.3 mm parasitic wasps to 80 mm walking sticks (Fig. 1A). We converted body length (mm) to dry body mass (mg) using equations derived from models based on large insect data sets (Table S2) [35]–[42]. Models were specific to insect order, thereby controlling for differences in body plan among taxonomic groups. For orders without models (Phasmatodea, Siphonaptera) we estimated body mass using general insect models. For most orders, multiple models were available (Table S2). Rather than attempting to identify the “best” model, we averaged dry mass estimates from all available models to obtain a consensus estimate. This ensemble approach should yield a lower mean error because the individual estimates come from models derived from independent data sets [43], [44].

Although based on the best available data for insect body sizes, indirectly estimating body mass from body length using these equations could add significant measurement error as suggested by the range of scaling exponents for the length-mass models (Table S2). We used a resampling approach to determine whether this source of error significantly altered the scaling relationships between body mass and development time. Independent length-mass models (Table S2) yielded 2–7 estimates of dry mass for each species, with 88% of species having 5 or more body mass estimates and 9% having only 2–3 estimates. The means and standard deviations of these dry mass estimates were used to generate random normal distributions of dry mass for each species. We then selected a single mass estimate for each species by random draw from their respective distributions and used these data in analyses of the scaling of development time with body mass (see below). This procedure was repeated 10,000 times to estimate the effects of mass measurement error on estimates of the scaling exponent of and variance explained by the scaling analysis.

We performed regression analyses using ordinary least squares (OLS), standardized major axis (SMA), and phylogenetic generalized least squares (PGLS) techniques. OLS analyses assume that the predictor variable (body mass) is measured without error so may underestimate allometric slopes if that is not the case [45], [46]. SMA analyses (model II or reduced major axis regression) assume equal measurement error in dependent and independent variables. SMA may therefore under- or overestimate slope values if the dependent or independent variables have more measurement error, respectively [46]. To account for the statistical non-independence of species data due to shared evolutionary history [47], we used PGLS which generalizes the phylogenetically independent contrasts (PICs) [47]–[49] method to deal with multichotomies and more complex models of evolution [50], [51]. For this analysis, we constructed a phylogeny for all taxa (Text S1) based on best available molecular and morphological data (Text S2), and assuming branch lengths of one given the lack of comparable branch length data across studies. From the phylogeny, a correlation structure was estimated for incorporation into a generalized least squares model [52]. We compared the performance of four evolutionary models that varied in the model of trait evolution: Ornstein-Uhlenbeck (OU) [53] vs. Brownian motion, and in branch lengths: Grafen’s transformation [48] vs. untransformed with all equal to 1. The OU model assumes that trait evolution is constrained, with the strength described by the parameter α [53], which was optimized by restricted maximum likelihood (REML) methods [52]. For each analysis, we chose from among these four models based on the lowest AIC value. We estimated R^2^ values for PGLS analyses following Paradis (2011) [52].

For comparison with other empirical studies, we first estimated the scaling of minimum development time (*T*_dev_) with dry body mass (both variables ln-transformed) among all insect species, using all three regression methods. For the OLS and SMA regressions, we used robust methods to minimize the effect of potential outliers on slope estimates [54], but these methods were not available for the PGLS analysis. We then performed an OLS regression with minimum development time as the response variable and dry body mass, *T*_opt_, insect order, and all interactions as the predictor variables. For comparison with the OLS full-factorial model, we ran a similar PGLS model that included mass, *T*_opt_, and insect order, but excluded the *T*_opt_ by insect order interaction because of singularities. In both cases, we removed non-significant interactions to obtain the final models. SMA methods were not available for models with covariate interactions. To visualize how optimal development time varied with *T*_opt_ and insect order, we used the final OLS model to predict development time (mean and standard error) at *T*_opt = _25 and 35°C for a 1 mg insect species in those orders with at least 10 species.

Analyses were performed in R [55] using the smatr package [56] for SMA and OLS analyses, and the caper [57] and nlme [58] packages for the PGLS analyses following the approach described by Paradis (2011) [52].

## Results

**Figure 2:**
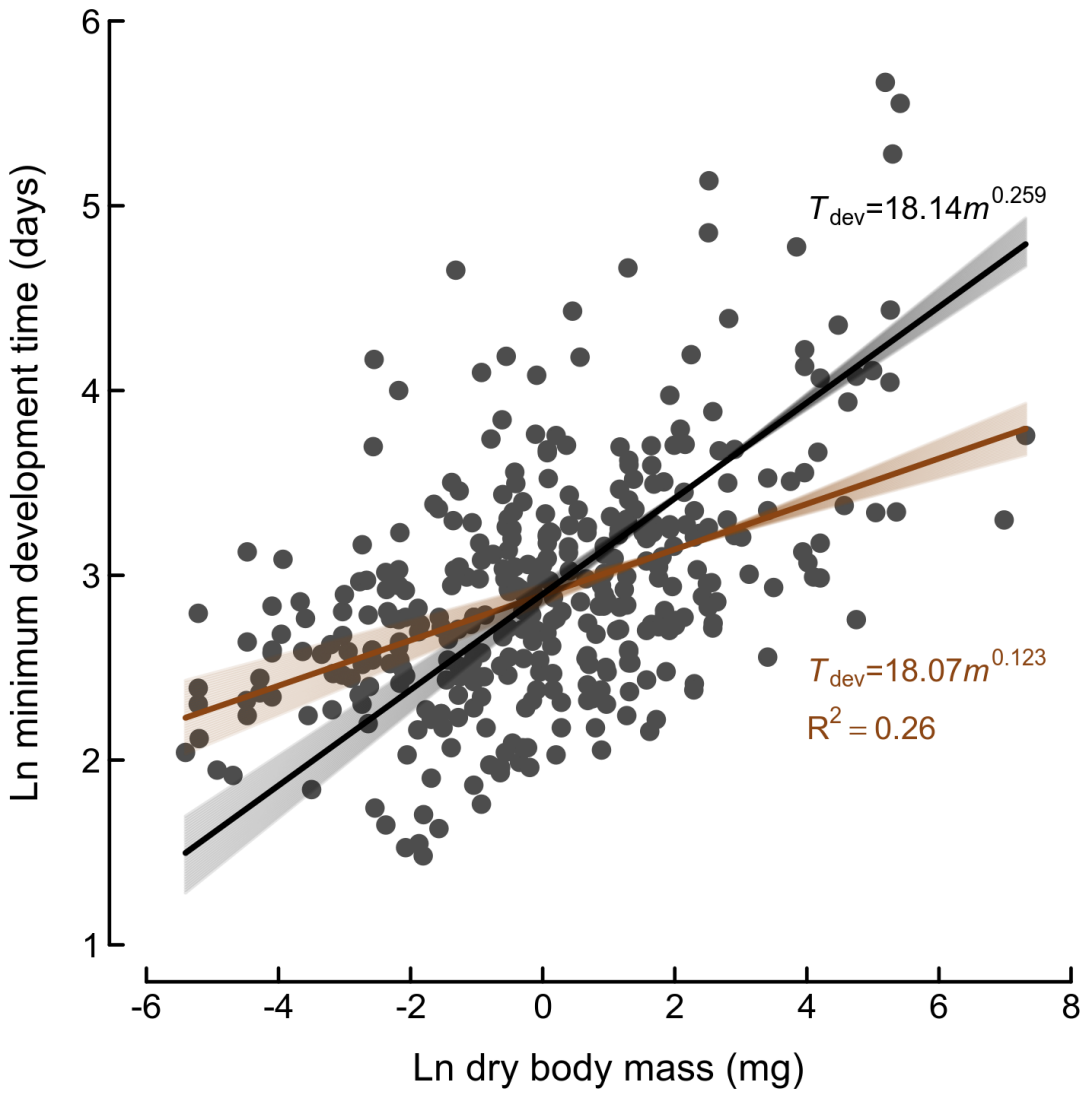
Scaling of minimum development time with body mass among insect species. The standardized major axis (black line) scaling exponent of 0.259 was not significantly different from 0.25 (95% CI: 0.234–0.287, shown in gray). The slope estimated by ordinary least squares (brown line) was significantly lower than 0.25 (95% CI: 0.096–0.149; R^2^ = 0.26; shown in light brown). Shading indicates 95% confidence intervals.

Among 361 insect species from 16 orders, spanning nearly 6 orders of magnitude in body mass (Table 1), development time increased significantly with body mass regardless of analytical approach. The SMA scaling exponent (0.259; Fig. 2, black line) was not significantly different from the predicted exponent of 0.25 (95% CI = 0.234–0.287; Fig. 2, black line). However, the OLS analysis yielded a slope of 0.123, which was significantly smaller than 0.25 (95% CI = 0.096–0.149; R^2^ = 0.26; Fig. 2, brown line). For the PGLS analysis, the best performing model assumed OU trait evolution with untransformed branch lengths (AIC = 461.3 vs. 468.1, 506.9, and 627.6 for the other three models). The allometric exponent from this PGLS analysis (0.097) was also significantly less than 0.25 (95% CI = 0.062–0.131, R^2^ = 0.28).

**Table 1:** Ranges for dry mass, optimal developmental temperature (*T*_opt_), and minimum development time (*T*_dev_) within the 16 insect orders and 361 insect species included in analyses.

Order	N (species)	Dry mass (mg)^1^	*T*_opt_ (°C)^2^	*T*_dev_ (days)^3^
Blattaria	2	16.7–179.1	30–33	80.6–289.4
Coleoptera	102	0.096–224.4	18–37.8	5.5–258.3
Collembola	4	0.078–0.666	15–28	31.1–104.7
Dermaptera	3	3.6–16.5	30–32.5	26.8–48.7
Diptera	42	0.019–13.1	20–36	7.1–59.3
Ephemeroptera	3	1.09–46.7	15–30	33.9–118.8
Hemiptera	86	0.025–66.8	19.5–36.7	4.4–83.9
Hymenoptera	63	0.004–22.8	23.9–35.7	6.3–36.5
Lepidoptera	18	1.497–211.9	24.3–33	12.8–40.6
Neuroptera	6	1.975–7.3	21.1–35	15.5–40.5
Odonata	2	192.1–200.2	24–25	57.1–196.4
Orthoptera	10	52.7–1082.5	30–40	15.8–84.4
Phasmatodea	1	1499.2	28	42.8
Psocodea	3	0.05–0.069	32.5–37.5	11.5–19.55
Siphonaptera	6	0.028–0.263	26–35	7.6–20.68
Thysanoptera	10	0.005–0.125	27.5–35	9.7–16.35

^1^ Order-specific consensus estimates from published equations relating dry mass to body length (see text and Table S2).

^2^ Temperature at which minimum egg to adult development time occurred (see text and Fig. 1).

^3^ Minimum egg to adult development time (see text).

Measurement error in body masses appears to have had little effect on model results. The scaling exponents and R^2^ values estimated for observed data (Fig. 2) closely matched 10,000 analyses of randomized data (Fig. S1) in which the body mass of each of the 361 species was randomly selected from a normal distribution with a mean and sd estimated from species-specific summarized mass body length equations (Table S2). The average (OLS) scaling exponent from the randomization approach was 0.119 (95% CI = 0.108–0.130) as compared to the observed (OLS) exponent of 0.123 (Fig. S1, A). R^2^ values from the randomization analysis averaged 0.22, with most of them (95% CI = 0.19–0.26) falling below the observed R^2^ of 0.26 (Fig. S1, B).

For the full-factorial OLS model including dry mass, *T*_opt_, and insect order, we removed the non-significant three-way interaction (mass: *T*_opt_:order; *F*_9,307_ = 0.94, *P* = 0.488), and the mass:order interaction (*F*_14,316_ = 1.36, *P* = 0.172) to generate the final model. Along with the main effects, the final, best-supported model (AIC = 479.8 vs 497.5 and 489.4 for models excluding the three-way or both the three-way and mass:order interaction, respectively) included *T*_opt_:order and *T*_opt_:mass interactions and explained 56% of the variation in *T*_dev_ (Table 2) Most of the variation in *T*_dev_ was explained by mass (26%) and insect order (16%), with *T*_opt_ and the two interactions explaining 5% and 4% each, respectively (Table 2). To verify that these results (in particular the order effects) were not a byproduct of low numbers of species in some orders (Table 1), we ran these models again including only the seven orders with 10 or more species (Table 1). As before, the three-way and mass:order interactions were not significant (*F*_6,303_ = 1.13, *P* = 0.342; *F*_6,309_ = 1.18, *P* = 0.319, respectively) so were not included in the final model. All other effects were strongly significant (all *P*<0.002). The final model with only these seven orders explained 46% of the variation in *T*_dev_, and as in the model including all orders, mass (25%) and order (9%) explained most of the variation in *T*_dev_.

**Table 2:** Among insects, minimum development time (*T*_dev_) varied with dry body mass, optimum developmental temperature (*T*_opt_), and insect order.

	OLS			PGLS^1^	
Effect	coefficient	95% CI	R^2^	coefficient	95% CI
Mass	0.772**	0.481, 1.06	0.26	0.398**	0.224, 0.572
*T*_opt_	−0.284**	−0.701, 0.135	0.05	−0.055**	−0.065, −0.044
Order	**	–	0.16	n.s.	–
mass:*T*_opt_	−0.022**	−0.032, −0.013	0.04	−0.011**	−0.017, −0.006
*T*_opt_:order^2^	*	–	0.04	–	–

^1^ The best performing PGLS model (AIC = 370, R^2^ = 0.54) was based on Brownian Motion trait evolution with branch lengths equal to 1, with REML-optimized α equal to 0.18. Methods for estimation of partial R^2^ from PGLS models were not available.

^2^ This interaction was excluded from the PGLS model because of singularities.

P<0.001, *P<0.01, n.s.: not significant.

For the PGLS analysis, the best performing model assumed Brownian evolution along untransformed branch lengths (AIC = 370.0 vs. 382.9, 413.0, and 484.2 for other models). The final model included mass, *T*_opt_, and the mass:*T*_opt_ interaction, and explained 54% of the variation in minimum development time (Table 2). In contrast to the OLS analyses, the PGLS analysis found no differences in *T*_dev_ among orders (Table 2), suggesting the phylogeny adequately captured variation in *T_dev_* at the order level.

**Figure 3:**
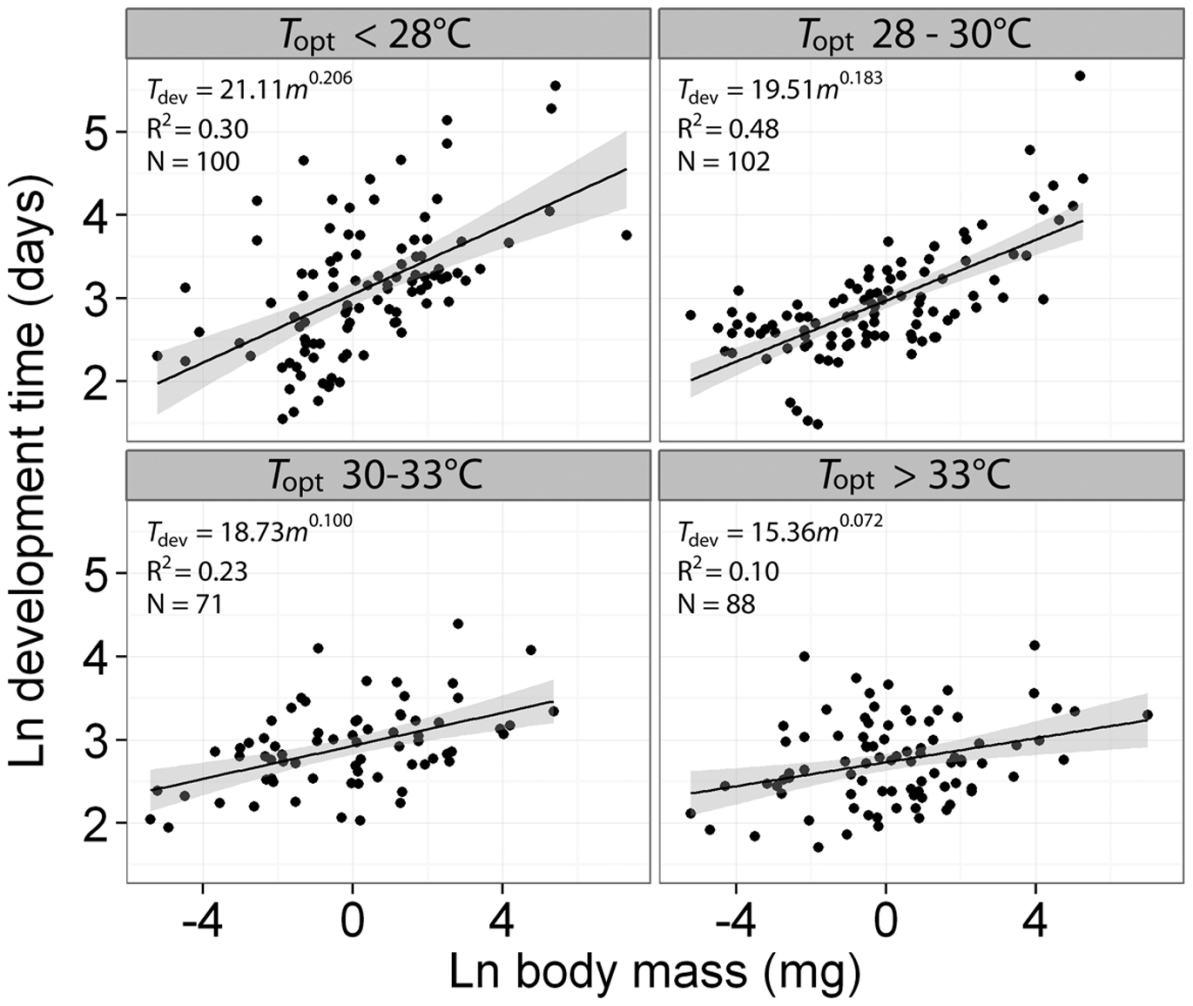
The scaling of minimum development time with body mass depends on optimal developmental temperature. Development time as a function of body mass (both ln-transformed) for four ranges of *T*_opt_. Insect species with lower *T*_opt_ showed steeper scaling of *T*_dev_ with body mass than did insect species with higher *T*_opt_ (scaling exponents fall from 0.206 to 0.072 as *T*_opt_ increased from less than 28 to over 33°C; Table 2, mass: *T*_opt_ interaction for both OLS and PGLS, *P*<0.001).

Both OLS and PGLS models suggested that insect species with higher *T*_opt_ developed more quickly for their body size (OLS and PGLS *T*_opt_ effect, both *P*<0.001; Table 2). Further, the relationship between *T*_dev_ and body mass depended strongly on *T*_opt_ (OLS and PGLS mass: *T*_opt_ interaction, both *P*<0.001; Table 2). Specifically, development time increased more steeply with mass for insect species with low *T*_opt_ than for species with high *T*_opt_ (Fig. 3). This interaction was not driven by covariance between *T*_opt_ and dry mass (OLS: *F*_1,344_ = 0.23, *P* = 0.636; PGLS: *t*_1,359_ = 0.37, *P* = 0.5451). SMA analyses of raw development time (order-corrected analyses were not possible with SMA) showed similar patterns, with the slope estimate decreasing from 0.400 (95% CI: 0.333, 0.480) for species with *T*_opt_ less than 28°C to 0.225 (95% CI: 0.190, 0.266) for species with *T*_opt_ between 28 and 30°C, to 0.192 (95% CI: 0.153, 0.241) for species with *T*_opt_ between 30 and 33°C. For species with *T*_opt_ greater than 33°C, the SMA slope estimate was 0.233 (95% CI: 0.187, 0.289).

Some insect orders developed more quickly than others after controlling for body size and *T*_opt_ (i.e. variation in intercept; OLS order effect, *P*<0.001; Table 2). However, the relationship between mass and *T*_dev_ did not differ among orders (OLS mass:order interaction, *P* = 0.172).

**Figure 4:**
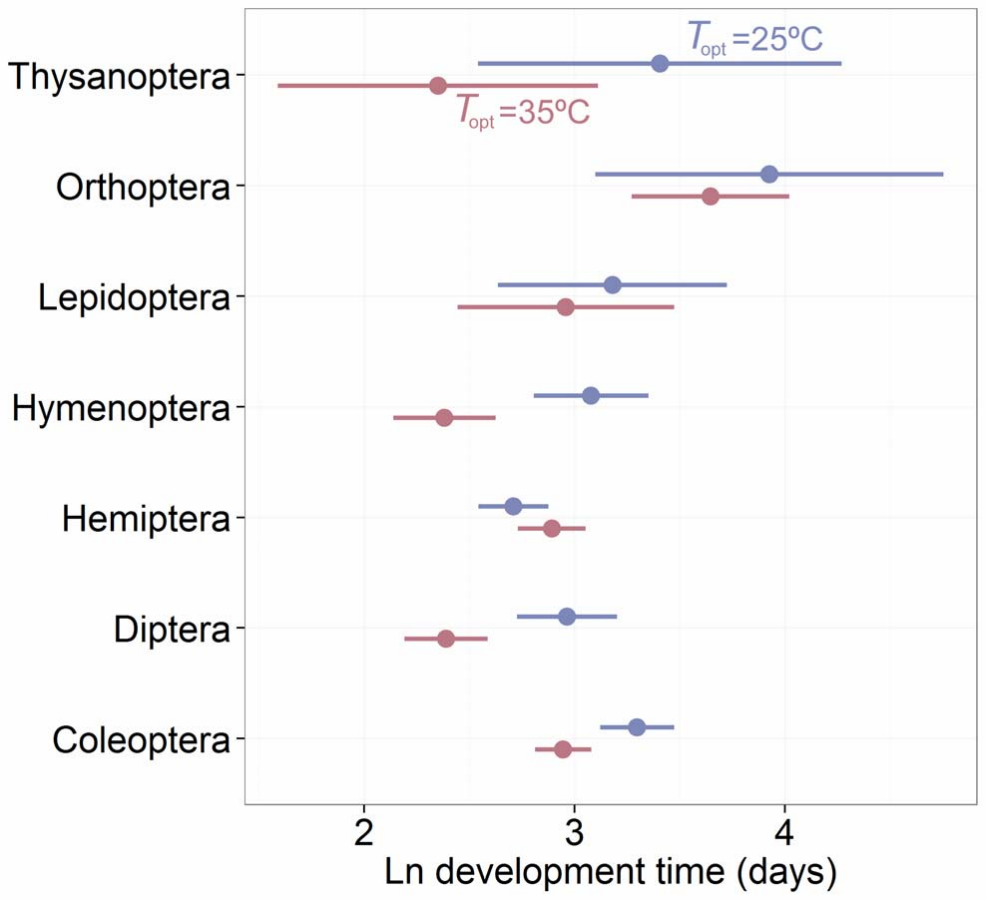
The effect of optimal developmental temperature on the scaling of development time varies among insect orders. Model-predicted (Table 2) development time (mean and 95% CI) for insects species corrected to 1 mg mass and *T*_opt_ values of 25 and 35°C. For some orders (Hymenoptera, Diptera, Coleoptera), species with higher *T*_opt_ developed much more quickly, whereas *T*_opt_ had little to no effect on development time in other orders.

The OLS analysis suggested that the relationship between *T*_dev_ and *T_o_*_pt_ differed among orders (OLS *T*_opt_:order interaction, *P*<0.01; Table 2). For most orders, after controlling for body size, species with higher *T*_opt_ developed more quickly (Fig. 4). This effect was strong for the Hymenoptera, Diptera, and Coleoptera (Fig. 4), but less pronounced or not evident for the Orthoptera, Lepidoptera, and Hemiptera when *T*_opt_ of 25 and 35°C were compared (Fig. 4). However, these results may be confounded by significant variation in *T*_opt_ among orders (OLS: *F*_15,344_ = 4.33, *P*<0.001; PGLS: *F*_1,15_ = 3.126, *P*<0.001; Fig. S2).

## Discussion

Across this large and diverse insect data set, body size was an unexpectedly weak predictor of development time, and we did not find strong support for quarter power scaling of development time with body mass as predicted by the ontogenetic growth model (OGM). When only body mass was included as a predictor, the scaling of minimum development time with body size varied with the analytical approach. The scaling exponents estimated by OLS and PGLS analyses were significantly smaller than 0.25 (0.123 and 0.097, respectively), but these approaches assume no error in the body mass estimates, so may underestimate the true scaling exponent [46]. The SMA analysis, which assumes equal error in estimates of body mass and minimum development time, yielded a scaling exponent that was indistinguishable from 0.25 (Fig. 2, Table 1), consistent with the OGM [12], [18], [19]. We don’t know the relative errors in estimates of *T*_dev_ and body mass, but errors in body mass estimates may be high because they include both measurement and equation error.

Length-mass relationships from diverse studies (Table S2) yielded large variation in body mass estimates for some species, creating a potentially significant source of error that might explain the relatively low scaling coefficients and poor predictive power of the regression models. However, the distribution of estimated exponents from a resampling simulation clearly overlapped the original estimate (Fig. S1), suggesting that error due to indirectly estimating body mass had little effect on slope estimates. This is likely because the error in body mass estimates was small relative to the range of body sizes included in the study. Although mass estimates clearly include error, the slope estimates from the regression analyses depend on relative error, which, given the large range in body masses, may be similar for the two axes. Consequently, the best estimate of the scaling exponent is likely between the OLS and SMA values.

The scaling of development time with body mass may differ from predictions because the assumptions of the OGM model are violated. One key assumption of the OGM is that whole organism metabolic rates scales with mass^0.75^. A number of studies suggest that metabolic scaling exponents can significantly deviate from the predicted ¾ power within and among species and across broader taxonomic groups [59]–[66]. However, two large scale studies in insects found that, after correcting for phylogenetic nonindependence, metabolic rate scaled with mass^0.75^ (391 insect species from 16 orders) [67], and with mass^0.76^ (419 species from 11 orders) [68]. Based on available data, it appears unlikely that the smaller exponents we found arise because of violation of this key assumption of the OGM. Limited evidence suggests that other assumptions of the OGM may also be violated, including invariance in energy metabolism among tissues [13] and constant cell size [69], but see [19] for further evaluation of some of these assumptions. Clearly, more studies testing the assumptions of the OGM are necessary to evaluate whether we expect its predictions of development time scaling to hold.

The scaling exponent may also differ from the predicted 0.25 power due to strong selection on development time for species with larger body sizes. The strong negative allometry of development time with body size described here (exponent between 0.12 and 0.25; Figs 2, 3) means that larger insects develop even more rapidly than expected from predictions based on bioenergetics alone [19], suggesting that there may be strong selective pressure for larger insects to develop more quickly.

Body size alone explained only 26–28% of the variation in development time, and there was approximately 10-fold variation in development time at any given body size (Fig. 2). Including the optimal temperature for development, *T*_opt_, in the PGLS model and *T*_opt_ and order in the OLS model doubled the amount of variation explained (Table 2). *T*_opt_ proved to be a strong predictor of minimum development time. Consistent with the “warmer is better” hypothesis [31], [32], we found that warm-adapted insects (i.e., those with higher *T*_opt_) had shorter development times at their optimal temperature than did cold-adapted species (i.e, those with lower *T*_opt_; Table 2, Fig. 4). This provides further evidence that although insects have adapted to a wide range of thermal environments, they do not fully compensate for the depressing effects of low temperatures on rates of biological processes [70]–[72], including development. The evolutionary adaptation of insects to their thermal environment therefore appears to be a particularly important factor driving minimum development times.

Beyond direct effects of optimal temperature on development time, the scaling relationship between body mass and development time varied with *T*_opt_ (Table 2, Fig. 3). For insects with low *T*_opt_ (i.e., those adapted to cold environments), development time increased relatively quickly with mass^0.21^; conversely, for insects with high *T*_opt_ (i.e., warm-adapted), development time increased only slightly with mass^0.07^ (Fig. 3). This temperature dependence of the scaling of development time with body size is contrary to one of the fundamental predictions of the MTE and OGM–a single slope of 0.25 for all insects, regardless of other factors [14], [15]. These findings suggest that, rather than following universal scaling, insects adapted to cold environments pay a larger cost (in terms of time) to develop larger body sizes, with potentially broad ecological implications. For insects in warm tropical regions, one of the costs of evolving larger body sizes (longer development time) may be mitigated by the evolution of higher optimal temperatures. Relaxation of this selective pressure may more readily allow for evolution of larger body sizes, which is consistent with the prevalence of the largest insects in warm tropical environments.

The effect of *T*_opt_ on development time was not consistent among insect orders (Table 2, OLS *T*_opt_ by order interaction). With the exception of the Lepidoptera, the effect of *T*_opt_ on development time was pronounced for holometabolous orders for which we had at least 10 species (Fig. 4; Coloeptera, Diptera, Hymenoptera). For hemimetabolous orders (Orthoptera, Hemiptera, and Thysanoptera), the effect of *T*_opt_ was weaker, or not evident. However, significant variation in *T*_opt_ among orders makes interpretation of these patterns problematic.

Even after accounting for body size and *T*_opt_ (and insect order for OLS analyses), a substantial amount of variation in development time was unexplained. Ecological differences among taxonomic groups may drive differences in metabolic rates among invertebrates [67], [73] (e.g. active hunters with higher metabolic rates than detritivores) [68] and other organisms [74]–[76], potentially leading to differences in the scaling of development time with body size. More studies investigating potential ecological drivers of development time among diverse insects are necessary to better understand what drives the substantial variation in development time documented here. Finally, predictive relationships for development time as a function of body mass may be useful to both basic and applied ecologists, but we urge caution. High variability in development time at a given body mass cannot be ignored and could lead to large prediction errors.

## Supporting Information

Figure S1 **Error in body mass estimates had little effect on allometric slope and R^2^ estimates.** From 10,000 OLS regression analyses where body masses of each of the 361 species were drawn from random normal distributions based on means and standard deviations of multiple estimates from length-mass equations (Table S2), A) the distribution of estimated allometric slopes overlapped the original estimate (0.123, red line) with the 95% CI of the resampling distribution (0.108, 0.130) similar to the 95% CI of the original slope estimate (0.096, 0.149). B) The R^2^ value of the original slope estimate (0.26) was higher than the mean of the resampled estimates (0.22).
(TIFF)

Figure S2 **Optimal developmental temperature varied among insect orders.** For six of the seven orders with 10 or more species, *T*_opt_ was centered around 30 °C. The Orthoptera tended to have higher *T*_opt_, with the mean near 35 °C.
(TIFF)

Table S1 **Compiled species data.**
(DOC)

Table S2 **Compiled equations for estimating dry mass.**
(DOC)

Text S1 **Newick tree for all insect species.**
(TXT)

Text S2 **Description of and references for phylogenetic tree construction.**
(DOC)
