# Supplementary figures and images for: Thermodynamics Constrains Allometric Scaling of Optimal Development Time in Insects
Source: PLoS One. 2013 Dec 31;8(12):e84308. doi: 10.1371/journal.pone.0084308 (PMC3877264; doi:10.1371/journal.pone.0084308)

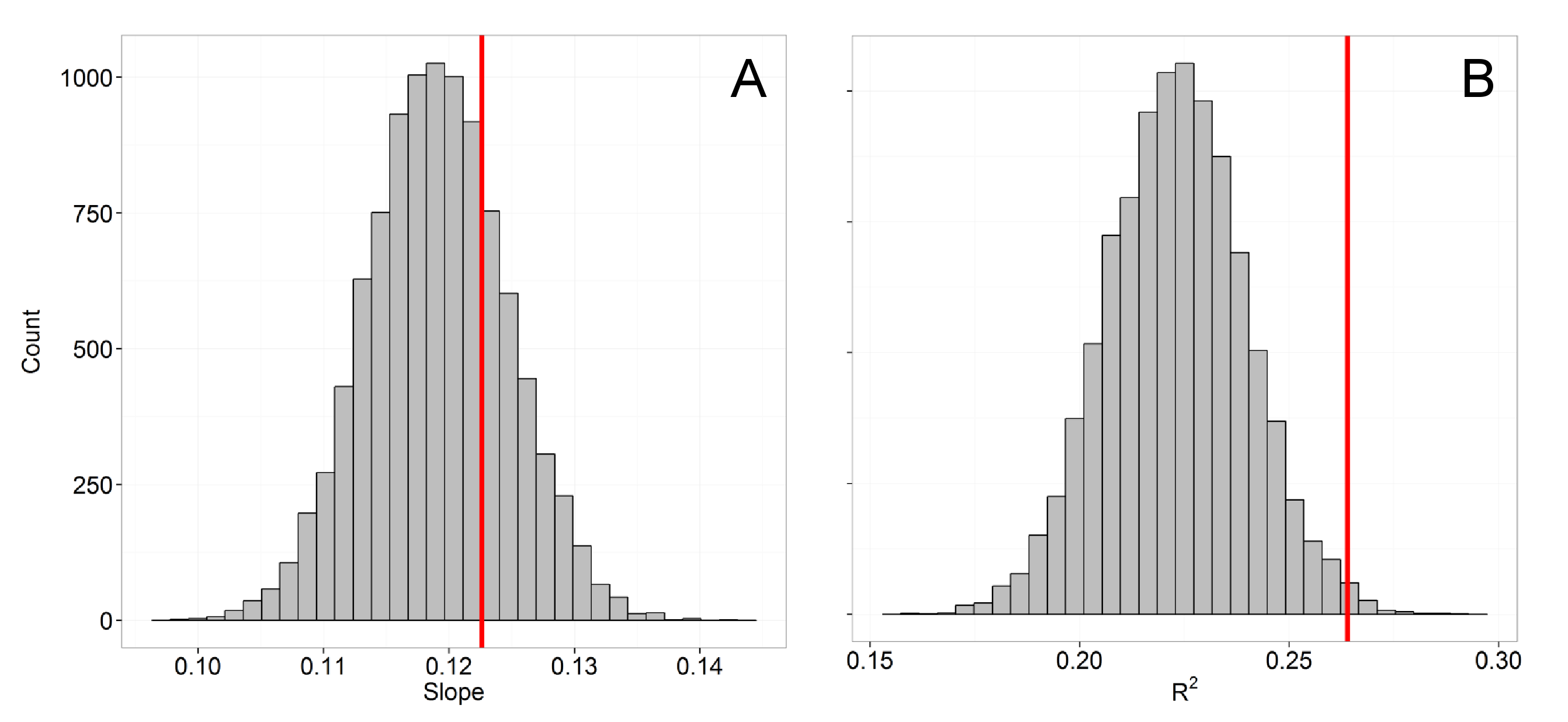

Supplement: Figure S1 — Error in body mass estimates had little effect on allometric slope and R2 estimates. From 10,000 OLS regression analyses where body masses of each of the 361 species were drawn from random normal distributions based on means and standard deviations of multiple estimates from length-mass equations (Table S2), A) the distribution of estimated allometric slopes overlapped the original estimate (0.123, red line) with the 95% CI of the resampling distribution (0.108, 0.130) similar to the 95% CI of the original slope estimate (0.096, 0.149). B) The R2 value of the original slope estimate (0.26) was higher than the mean of the resampled estimates (0.22). (TIFF) [file pone.0084308.s001.tiff]

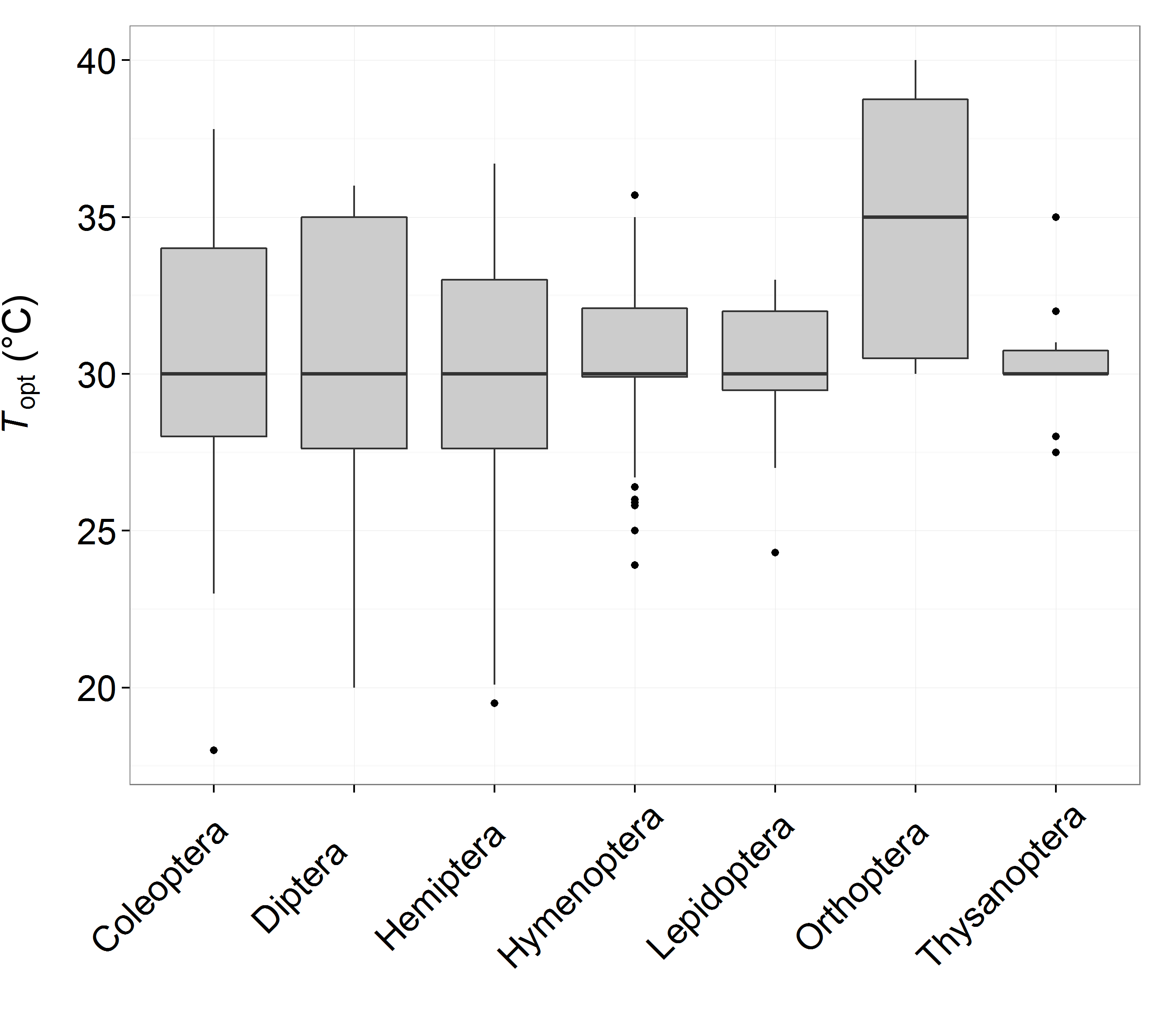

Supplement: Figure S2 — Optimal developmental temperature varied among insect orders. For six of the seven orders with 10 or more species, T opt was centered around 30 °C. The Orthoptera tended to have higher T opt, with the mean near 35 °C. (TIFF) [file pone.0084308.s002.tiff]
