# Supplementary material for: Thermodynamics Constrains Allometric Scaling of Optimal Development Time in Insects
Source: PLoS One. 2013 Dec 31;8(12):e84308. doi: 10.1371/journal.pone.0084308 (PMC3877264; doi:10.1371/journal.pone.0084308)
Supplement: Table S1 — Compiled species data. (DOC) [file pone.0084308.s003.doc]

**Table S1.** Minimum egg to adult development time (*T*_dev_), the temperature at which *T*_dev_ occurred (*T*_opt_), the number of available estimates of development time at different temperatures (N), and estimated dry mass for insect species included in the study (arranged alphabetically by order then by species, with family also given).

| **(order) species** | **family** | ***T*_dev_**  **(days)** | ***T*_opt_**  **(°C)** | **N** | **Body length**  **(mm)** | **dry mass**  **(mg)** | **reference** |
| --- | --- | --- | --- | --- | --- | --- | --- |
| **Blattaria** |  |  |  |  |  |  |  |
| *Periplaneta fuliginosa* | Blattidae | 289.4 | 30 | 5 | 33 | 179.11 | [1] |
| *Supella longipalpa* | Blatellidae | 80.6 | 33 | 3 | 12 | 16.72 | [2] |
| **Coleoptera** |  |  |  |  |  |  |  |
| *Adalia bipunctata* | Coccinelidae | 13 | 29.4 | 6 | 4.5 | 1.97 | [3] |
| *Ahasverus advena* | Sylvanidae | 16 | 30 | 7 | 2.2 | 0.35 | [4] |
| *Alphitobius diaperinus* | Tenebrionidae | 28.7 | 35 | 5 | 6.05 | 4.04 | [5] |
| *Amara aenea* | Carabidae | 25.8 | 27.2 | 4 | 7.5 | 6.85 | [6] |
| *Amara familiaris* | Carabidae | 21.7 | 27.2 | 4 | 6.5 | 4.82 | [6] |
| *Amara littorea* | Carabidae | 26.4 | 27.5 | 3 | 8.3 | 8.80 | [6] |
| *Amara ovata* | Carabidae | 26 | 27.1 | 4 | 9.5 | 12.30 | [6] |
| *Amara similata* | Carabidae | 25.2 | 27.2 | 4 | 9 | 10.76 | [6] |
| *Anoplophora glabripennis* | Cerambycidae | 258.3 | 25 | 4 | 30 | 224.39 | [7] |
| *Anthonomus eugenii* | Curculionidae | 12.9 | 30 | 7 | 2.75 | 0.60 | [8] |
| *Anthonomus grandis thurberiae* | Curculionidae | 17.1 | 30 | 5 | 5.75 | 3.57 | [9] |
| *Anthonomus signatus* | Curculionidae | 15 | 30 | 3 | 3 | 0.74 | [10] |
| *Aubeonymus mariaefranciscae* | Curculionidae | 65.4 | 26 | 4 | 4.3 | 1.76 | [11] |
| *Bagous affinis* | Curculionidae | 13.7 | 32 | 6 | 3.6 | 1.15 | [12] |
| *Baris lepidii* | Curculionidae | 43.1 | 27 | 6 | 3.25 | 0.90 | [13] |
| *Callosobruchus chinensis* | Chrysomelidae | 22 | 30 | 3 | 3.5 | 1.07 | [14] |
| *Callosobruchus maculatus* | Chrysomelidae | 23.9 | 35 | 3 | 3.5 | 1.07 | [14] |
| *Callosobruchus rhodesianus* | Chrysomelidae | 28 | 30 | 4 | 3.46 | 1.04 | [14] |
| *Callosobruchus subinnotatus* | Chrysomelidae | 25 | 35 | 3 | 5.47 | 3.16 | [15] |
| *Calvia quatuordecimguttata* | Coccinelidae | 14.6 | 30 | 5 | 4.75 | 2.24 | [16] |
| *Carpophilus dimidiatus* | Nitidulidae | 29.5 | 32.5 | 4 | 1.7 | 0.19 | [17] |
| *Cassida rubiginosa* | Chrysomelidae | 19.8 | 32.5 | 4 | 7 | 5.78 | [18] |
| *Cerotoma arcuatus* | Chrysomelidae | 22.7 | 32 | 7 | 4 | 1.48 | [19] |
| *Chilocorus nigritus* | Coccinelidae | 25.2 | 30 | 6 | 3.6 | 1.15 | [20] |
| *Cionus latefasciatus* | Curculionidae | 15.4 | 30 | 5 | 6.7 | 5.19 | [21] |
| *Coccinella novemnotata* | Coccinelidae | 10.7 | 32.2 | 4 | 5.85 | 3.73 | [22] |
| *Coccinella septempunctata* | Coccinelidae | 22.2 | 23 | 4 | 7.15 | 6.09 | [23] |
| *Coccinella transversoguttata* | Coccinelidae | 12.5 | 29.4 | 5 | 5.9 | 3.80 | [24] |
| *Coccinella trifasciata* | Coccinelidae | 11.1 | 34 | 5 | 4.5 | 1.97 | [25] |
| *Coleomegilla maculata lengi* | Coccinelidae | 14.9 | 27.3 | 5 | 5.4 | 3.06 | [26] |
| *Collops vittatus* | Melyridae | 33.8 | 32.2 | 6 | 6 | 3.96 | [27] |
| *Conotrachelus nenuphar* | Curculionidae | 32 | 28.7 | 6 | 5.5 | 3.20 | [28] |
| *Crioceris asparagi* | Chrysomelidae | 14.9 | 33 | 9 | 6.5 | 4.82 | [29] |
| *Cryptolestes ferrugineus* | Cucujidae | 21 | 37.5 | 9 | 2 | 0.28 | [30] |
| *Cryptolestes pusillus* | Cucujidae | 28.8 | 37.5 | 8 | 1.75 | 0.21 | [31] |
| *Cryptolestes turcicus* | Cucujidae | 31.7 | 32.5 | 5 | 2 | 0.28 | [32] |
| *Cylas formicarius elegantulus* | Brentidae | 32.9 | 27 | 4 | 6.8 | 5.39 | [33] |
| *Cylas puncticollis* | Brentidae | 15.2 | 35.8 | 5 | 7 | 5.78 | [34] |
| *Dendroctonus ponderosae* | Curculionidae | 30.2 | 25 | 3 | 5.82 | 3.68 | [35] |
| *Dermestes ater* | Dermestidae | 44.3 | 30 | 3 | 8 | 8.04 | [36] |
| *Dermestes frischii* | Dermestidae | 26.4 | 35 | 4 | 7.5 | 6.85 | [37] |
| *Dermestes haemorrhoidalis* | Dermestidae | 40.8 | 30 | 4 | 8.24 | 8.65 | [38] |
| *Dermestes maculatus* | Dermestidae | 36.4 | 35 | 4 | 6.71 | 5.21 | [39] |
| *Diomus austrinus* | Coccinelidae | 14.8 | 30 | 3 | 1.54 | 0.15 | [40] |
| *Diorhabda elongata* | Chrysomelidae | 19.97 | 35 | 4 | 5.75 | 3.57 | [41] |
| *Epilachna varivestis* | Coccinelidae | 24.5 | 27.3 | 5 | 6.5 | 4.82 | [42] |
| *Euhrychiopsis lecontei* | Curculionidae | 16.6 | 29 | 8 | 3 | 0.74 | [43] |
| *Galerucella calmariensis* | Chrysomelidae | 23.43 | 27.5 | 4 | 4 | 1.48 | [44] |
| *Galerucella pusilla* | Chrysomelidae | 24.73 | 27.5 | 4 | 3.5 | 1.07 | [44] |
| *Gastrophysa viridula* | Chrysomelidae | 15 | 28 | 4 | 5.5 | 3.20 | [45] |
| *Glischrochilus quadrisignatus* | Nitidulidae | 27.6 | 30 | 3 | 5.2 | 2.79 | [46] |
| *Gnathocerus maxillosus* | Tenebrionidae | 39.6 | 30 | 6 | 3.5 | 1.07 | [47] |
| *Gonipterus scutellatus* | Curculionidae | 53.2 | 26.8 | 5 | 7.5 | 6.85 | [48] |
| *Harmonia axyridis* | Coccinelidae | 11.4 | 34 | 6 | 6.5 | 4.82 | [16] |
| *Hippodamia convergens* | Coccinelidae | 16.9 | 23 | 4 | 5.5 | 3.20 | [23] |
| *Hippodamia parenthesis* | Coccinelidae | 11.9 | 30 | 5 | 5.09 | 2.65 | [49] |
| *Hippodamia sinuata* | Coccinelidae | 12.2 | 35 | 5 | 5.05 | 2.60 | [50] |
| *Hylobius pales* | Curculionidae | 66.3 | 28 | 3 | 8.55 | 9.47 | [51] |
| *Hypera brunneipennis* | Curculionidae | 22.5 | 26.7 | 4 | 5 | 2.54 | [52] |
| *Hypera meles* | Curculionidae | 17.4 | 37 | 8 | 4.31 | 1.77 | [53] |
| *Hypera postica* | Curculionidae | 10 | 35 | 8 | 5.07 | 2.63 | [54] |
| *Hyperaspis notata* | Coccinelidae | 21.2 | 32 | 6 | 3.4 | 1.00 | [55] |
| *Ips avulsus* | Curculionidae | 7.9 | 32 | 5 | 3 | 0.74 | [56] |
| *Ips calligraphus* | Curculionidae | 18.1 | 35 | 6 | 5 | 2.54 | [57] |
| *Ips confusus* | Curculionidae | 10.8 | 35 | 5 | 4.9 | 2.42 | [58] |
| *Ips typographus* | Curculionidae | 17 | 30 | 3 | 4.85 | 2.36 | [59] |
| *Laricobius nigrinus* | Derodontidae | 46.6 | 18 | 4 | 2.63 | 0.54 | [60] |
| *Latheticus oryzae* | Tenebrionidae | 18.4 | 37.5 | 4 | 2.75 | 0.60 | [61] |
| *Leptinotarsa decemlineata* | Chrysomelidae | 17.4 | 32 | 10 | 10 | 13.97 | [62] |
| *Lioadalia flavomaculata* | Coccinelidae | 9.4 | 32 | 6 | 5.75 | 3.57 | [63] |
| *Listronotus oregonensis* | Curculionidae | 27.2 | 32.3 | 6 | 5.75 | 3.57 | [64] |
| *Monochamus carolinensis* | Cerambycidae | 51.3 | 30 | 3 | 22 | 101.58 | [65] |
| *Nephaspis oculatus* | Coccinelidae | 16.5 | 31 | 5 | 1.26 | 0.09 | [66] |
| *Oryzaephilus acuminatus* | Sylvanidae | 19.5 | 32.5 | 7 | 3.55 | 1.11 | [67] |
| *Oryzaephilus surinamensis* | Sylvanidae | 20.8 | 35 | 6 | 2.65 | 0.55 | [68] |
| *Otiorrhynchus sulcatus* | Curculionidae | 128.2 | 24 | 4 | 9.5 | 12.30 | [69] |
| *Oulema melanopus* | Chrysomelidae | 20.3 | 30 | 9 | 5 | 2.54 | [70] |
| *Palorus foveicollis* | Tenebrionidae | 40.6 | 32.5 | 6 | 3.95 | 1.44 | [71] |
| *Palorus laesicollis* | Tenebrionidae | 65.7 | 25 | 5 | 2.7 | 0.58 | [71] |
| *Palorus ratzeburgii* | Tenebrionidae | 26.1 | 33.8 | 8 | 2.7 | 0.58 | [71] |
| *Palorus subdepressus* | Tenebrionidae | 35.1 | 35 | 7 | 2.85 | 0.66 | [71] |
| *Popillia japonica* | Scarabaeidae | 169.9 | 25 | 3 | 9.53 | 12.40 | [72] |
| *Propylea dissecta* | Coccinelidae | 10.3 | 35 | 5 | 4.65 | 2.13 | [73] |
| *Prostephanus truncatus* | Bostrichidae | 25.4 | 32 | 9 | 3.55 | 1.12 | [74] |
| *Pterohelaeus alternatus* | Tenebrionidae | 68.1 | 28.6 | 4 | 17 | 52.83 | [75] |
| *Pterohelaeus darlingensis* | Tenebrionidae | 62.2 | 33.1 | 4 | 17 | 52.83 | [75] |
| *Pyrrhalta luteola* | Chrysomelidae | 21 | 32.2 | 4 | 7 | 5.78 | [76] |
| *Rhizopertha dominica* | Bostrichidae | 24.5 | 34 | 4 | 2.8 | 0.63 | [77] |
| *Rhyzobius lophanthae* | Coccinelidae | 23.9 | 30 | 4 | 2.28 | 0.39 | [78] |
| *Rodolia cardinalis* | Coccinelidae | 18.4 | 26 | 4 | 3.2 | 0.86 | [79] |
| *Sitophilus oryzae* | Curculionidae | 26.1 | 28 | 5 | 4.5 | 1.97 | [80] |
| *Stegobium paniceum* | Anobiidae | 42.8 | 27.5 | 7 | 3.7 | 1.23 | [81] |
| *Stethorus punctillum* | Coccinelidae | 5.5 | 35 | 9 | 1.59 | 0.17 | [82] |
| *Tribolium castaneum* | Tenebrionidae | 20.1 | 35 | 7 | 3.34 | 0.96 | [83] |
| *Tribolium confusum* | Tenebrionidae | 25 | 32.5 | 8 | 3.5 | 1.07 | [84] |
| *Tribolium freemani* | Tenebrionidae | 23.5 | 25 | 7 | 5 | 2.54 | [85] |
| *Tribolium madens* | Tenebrionidae | 25.3 | 35 | 7 | 4.49 | 1.96 | [86] |
| *Trogoderma anthrenoides* | Dermestidae | 42 | 35 | 5 | 2.45 | 0.46 | [87] |
| *Trogoderma glabrum* | Dermestidae | 29.9 | 35 | 4 | 3 | 0.74 | [88] |
| *Trogoderma variabile* | Dermestidae | 28.6 | 37.8 | 6 | 4.25 | 1.71 | [89] |
| *Trogoderma versicolor* | Dermestidae | 39 | 35 | 5 | 3.5 | 1.07 | [90] |
| *Typhaea stercorea* | Mycetophagidae | 25.8 | 30 | 5 | 2.8 | 0.63 | [91] |
| **Collembola** |  |  |  |  |  |  |  |
| *Folsomia candida* | Isotomidae | 31.1 | 22 | 4 | 2.3 | 0.55 | [92] |
| *Hypogastrura tullbergi* | Hypogastruridae | 104.7 | 15 | 3 | 1.7 | 0.27 | [93] |
| *Paronychiurus kimi* | Onychiuridae | 33 | 28 | 5 | 2.5 | 0.67 | [94] |
| *Protaphorura armatus* | Onychiuridae | 64.6 | 26 | 3 | 1 | 0.08 | [95] |
| **Dermaptera** |  |  |  |  |  |  |  |
| *Diaperasticus erythrocephala* | Forficulidae | 26.8 | 32 | 3 | 9 | 3.62 | [96] |
| *Euborellia annulipes* | Anisolabididae | 48.7 | 30 | 6 | 14 | 13.15 | [97] |
| *Nala lividipes* | Labiduridae | 33.1 | 32.5 | 4 | 15 | 16.48 | [98] |
| **Diptera** |  |  |  |  |  |  |  |
| *Aedes albopictus* | Culicidae | 14.9 | 25 | 5 | 4.5 | 0.91 | [99] |
| *Aedes campestris* | Culicidae | 10.1 | 27.5 | 4 | 5.25 | 1.33 | [100] |
| *Anopheles gambiae ss* | Culicidae | 9.8 | 28 | 8 | 4.2 | 0.77 | [101] |
| *Anopheles quadrimaculatus* | Culicidae | 8.8 | 34.8 | 7 | 5.25 | 1.33 | [102] |
| *Aphidoletes aphidimyza* | Cecidomyiidae | 15 | 25 | 3 | 2.75 | 0.28 | [103] |
| *Atherigona soccata* | Muscidae | 19.59 | 30 | 3 | 4.5 | 0.91 | [104] |
| *Bactrocera cucurbitae a* | Tephritidae | 11.92 | 36 | 6 | 10.1 | 6.48 | [105] |
| *Bactrocera dorsalis a* | Tephritidae | 13.4 | 34 | 7 | 8 | 3.68 | [105] |
| *Bactrocera tau* | Tephritidae | 13.28 | 28 | 3 | 8 | 3.68 | [106] |
| *Bradysia impatiens* | Sciaridae | 19.9 | 29.4 | 5 | 2.9 | 0.32 | [107] |
| *Calliphora vicina* | Calliphoridae | 18.9 | 23.3 | 3 | 10.5 | 7.12 | [108] |
| *Ceratitis capitata* | Tephritidae | 16.8 | 26 | 5 | 4.3 | 0.82 | [109] |
| *Ceratitis cosyra* | Tephritidae | 17.7 | 26 | 5 | 4.4 | 0.87 | [109] |
| *Ceratitis rosa* | Tephritidae | 17.8 | 26 | 5 | 5 | 1.18 | [109] |
| *Chironomus tepperi* | Chironomidae | 7.6 | 32.5 | 11 | 5.08 | 1.22 | [110] |
| *Contarinia nasturtii* | Cecidomyiidae | 18.6 | 30 | 3 | 1.75 | 0.09 | [111] |
| *Contarinia sorghicola* | Cecidomyiidae | 11.8 | 34 | 10 | 1.25 | 0.04 | [112] |
| *Culex annulirostris* | Culicidae | 7.1 | 35 | 4 | 4.31 | 0.82 | [113] |
| *Culex tarsalis* | Culicidae | 10.8 | 35 | 5 | 4.5 | 0.91 | [114] |
| *Culicoides variipennis* | Ceratopogonidae | 14.6 | 30 | 5 | 0.9 | 0.02 | [115] |
| *Cystiphora schmidti* | Cecidomyiidae | 13.8 | 30 | 4 | 1.23 | 0.04 | [116] |
| *Delia florilega* | Anthomyiidae | 19 | 30 | 4 | 6.75 | 2.44 | [117] |
| *Delia platura* | Anthomyiidae | 17 | 35 | 5 | 6.84 | 2.52 | [117] |
| *Drosophila melanogaster* | Drosophilidae | 8.8 | 26 | 4 | 2.5 | 0.22 | [118] |
| *Feltiella acarisuga* | Cecidomyiidae | 10 | 27.5 | 4 | 1.5 | 0.07 | [119] |
| *Haematobia irritans exigua* | Muscidae | 7.9 | 35 | 5 | 4.25 | 0.80 | [120] |
| *Hippelates bishoppi* | Chloropidae | 13.4 | 35 | 4 | 1.6 | 0.08 | [121] |
| *Hippelates pallipes* | Chloropidae | 12.7 | 35 | 4 | 1.6 | 0.08 | [121] |
| *Hippelates pusio* | Chloropidae | 13.4 | 35 | 4 | 1.6 | 0.08 | [121] |
| *Liriomyza trifolii* | Agromyzidae | 14 | 35 | 5 | 1.9 | 0.11 | [122] |
| *Lucilia sericata* | Calliphoridae | 10.8 | 34 | 9 | 12 | 9.86 | [123] |
| *Mayetiola destructor* | Cecidomyiidae | 26.7 | 23.9 | 5 | 3 | 0.34 | [124] |
| *Musca autumnalis* | Muscidae | 7.8 | 34.7 | 7 | 6.75 | 2.44 | [125] |
| *Musca domestica* | Muscidae | 8.8 | 35 | 7 | 6.5 | 2.22 | [126] |
| *Ophyra aenescens* | Muscidae | 12.4 | 30 | 3 | 6.25 | 2.02 | [127] |
| *Parasarcophaga ruficornis* | Sarcophagidae | 15.1 | 34 | 7 | 13.5 | 13.13 | [128] |
| *Phormia regina* | Calliphoridae | 16.2 | 35 | 5 | 10 | 6.33 | [129] |
| *Piophila casei* | Piophilidae | 14.7 | 32 | 5 | 4.8 | 1.07 | [130] |
| *Protophormia terraenovae* | Calliphoridae | 9.2 | 35 | 5 | 9.5 | 5.59 | [131] |
| *Psila rosae* | Psilidae | 59.3 | 20 | 4 | 4.5 | 0.91 | [132] |
| *Sepedon fuscipennis* | Sciomyzidae | 16.6 | 30 | 6 | 10 | 6.33 | [133] |
| *Stomoxys calcitrans* | Muscidae | 12.5 | 30 | 5 | 8 | 3.68 | [134] |
| **Ephemeroptera** |  |  |  |  |  |  |  |
| *Cloeon triangulifer* | Baetidae | 33.9 | 25 | 3 | 5.9 | 1.09 | [135] |
| *Ephemerella subvaria* | Ephemerellidae | 106 | 15 | 2 | 9 | 3.64 | [136] |
| *Hexagenia bilineata* | Ephemeridae | 118.8 | 30 | 4 | 22 | 46.73 | [137] |
| **Hemiptera, Auchenorrhyncha** |  |  |  |  |  |  |  |
| *Acrosternum hilare* | Pentatomidae | 40.3 | 27 | 5 | 1.6 | 0.08 | [138] |
| *Anasa tristis* | Coreidae | 23.7 | 33.3 | 7 | 1.5 | 0.07 | [139] |
| *Apolygus lucorum* | Miridae | 10.2 | 30 | 6 | 5.5 | 1.98 | [140] |
| *Biprorulus bibax* | Pentatomidae | 23.9 | 32.5 | 6 | 20 | 66.84 | [141] |
| *Cermatulus nasalis* | Pentatomidae | 19.8 | 30 | 3 | 20 | 66.84 | [142] |
| *Cicadulina mbila* | Cicadellidae | 19.7 | 30.1 | 11 | 2.5 | 0.38 | [143] |
| *Cicadulina parazeae* | Cicadellidae | 19 | 19 | 3 | 2.1 | 0.25 | [143] |
| *Cicadulina storeyi* | Cicadellidae | 19.7 | 19.7 | 3 | 2.94 | 0.56 | [143] |
| *Empoasca fabae* | Cicadellidae | 14.4 | 29.9 | 4 | 2.9 | 0.55 | [144] |
| *Eutettix tenellus* | Cicadellidae | 18.6 | 35 | 11 | 3.25 | 0.72 | [145] |
| *Graminella nigrifrons* | Cicadellidae | 21.3 | 30 | 5 | 3.4 | 0.80 | [146] |
| *Ommatissus lybicus* | Tropiduchidae | 83.9 | 27.5 | 6 | 4.5 | 1.57 | [147] |
| **Hemiptera, Heteroptera** |  |  |  |  |  |  |  |
| *Clavigralla shadabi* | Coreidae | 15.73 | 34 | 8 | 9 | 7.46 | [148] |
| *Clavigralla tomentosicollis* | Coreidae | 11.17 | 34 | 7 | 10 | 9.94 | [148] |
| *Corythucha cydoniae* | Tingidae | 20.1 | 31.7 | 3 | 3.5 | 0.60 | [149] |
| *Creontiades dilutus* | Miridae | 14.9 | 31 | 5 | 8.7 | 6.81 | [150] |
| *Deraeocoris brevis* | Miridae | 15.9 | 32 | 4 | 4.6 | 1.23 | [151] |
| *Dicyphus hesperis* | Miridae | 15.7 | 35 | 5 | 4.5 | 1.16 | [152] |
| *Euschistus conspersus* | Pentatomidae | 24.7 | 32.5 | 3 | 10 | 9.94 | [153] |
| *Geocoris atricolor* | Lygaeidae | 18.6 | 35 | 5 | 3.5 | 0.60 | [154] |
| *Geocoris pallens* | Lygaeidae | 16.2 | 35 | 5 | 4 | 0.85 | [154] |
| *Gerris buenoi* | Gerridae | 27.1 | 26 | 3 | 12 | 16.35 | [155] |
| *Gerris comatus* | Gerridae | 28.5 | 26 | 4 | 15 | 30.17 | [155] |
| *Gerris pingreensis* | Gerridae | 24.7 | 26 | 4 | 13 | 20.36 | [155] |
| *Halyomorpha halys* | Pentatomidae | 33.4 | 30 | 6 | 17 | 42.62 | [156] |
| *Ischnodemus variegatus* | Blissidae | 40.23 | 30.5 | 6 | 6.6 | 3.23 | [157] |
| *Joppeicus paradoxus* | Joppeicidae | 60.2 | 32 | 5 | 3 | 0.40 | [158] |
| *Lyctocoris campestris* | Anthocoridae | 28.3 | 29 | 4 | 3.6 | 0.64 | [159] |
| *Lygus elisus* | Miridae | 10.8 | 35 | 6 | 4.52 | 1.17 | [160] |
| *Lygus hesperus* | Miridae | 16.5 | 35 | 5 | 4.75 | 1.34 | [161] |
| *Lygus lineolaris* | Miridae | 19.7 | 28 | 4 | 5.45 | 1.93 | [162] |
| *Macrolophus pygmaeus* | Miridae | 23 | 27.5 | 5 | 3.5 | 0.60 | [163] |
| *Nabis americoferus* | Nabidae | 18.6 | 33 | 6 | 6.76 | 3.44 | [164] |
| *Nabis capsiformis* | Nabidae | 19.3 | 28 | 3 | 11 | 12.89 | [165] |
| *Nysius vinitor* | Lygaeidae | 15.1 | 35 | 7 | 3.5 | 0.60 | [166] |
| *Oebalus pugnax* | Pentatomidae | 20.7 | 30 | 4 | 10.16 | 10.38 | [167] |
| *Oechalia schellenbergii* | Pentatomidae | 12.9 | 35 | 4 | 15 | 30.17 | [142] |
| *Orius albidipennis* | Anthocoridae | 10.4 | 35 | 2 | 3 | 0.40 | [168] |
| *Orius insidiosus* | Anthocoridae | 12.1 | 32 | 4 | 1.9 | 0.12 | [169] |
| *Orius laevigatus* | Anthocoridae | 13.2 | 35 | 3 | 3 | 0.40 | [168] |
| *Orius sauteri* | Anthocoridae | 12.7 | 30 | 4 | 2.47 | 0.24 | [170] |
| *Orius strigicollis* | Anthocoridae | 11.4 | 30 | 4 | 2.45 | 0.23 | [171] |
| *Orius tristicolor* | Anthocoridae | 12.4 | 35 | 4 | 1.5 | 0.07 | [172] |
| *Podisus acutissimus* | Pentatomidae | 15.3 | 35 | 5 | 9 | 7.46 | [173] |
| *Podisus maculiventris* | Pentatomidae | 19.2 | 35 | 11 | 10.75 | 12.11 | [174] |
| *Podisus nigrispinus* | Pentatomidae | 15.5 | 33 | 7 | 11.1 | 13.21 | [175] |
| *Podisus sagitta* | Pentatomidae | 16.9 | 33 | 5 | 10.8 | 12.26 | [176] |
| *Pristhesancus plagipennis* | Reduviidae | 58.4 | 30 | 4 | 20 | 66.84 | [177] |
| *Pseudatomoscelis seriatus* | Miridae | 8.8 | 35 | 5 | 3.08 | 0.42 | [178] |
| *Pyrrhocoris apterus* | Pyrrhocoridae | 28.48 | 28 | 5 | 10 | 9.94 | [179] |
| *Rhinacloa forticornis* | Miridae | 15.9 | 30 | 3 | 1.77 | 0.10 | [180] |
| *Riptortus clavatus* | Alydidae | 18.8 | 36.7 | 7 | 15.5 | 33.03 | [181] |
| *Stephanitis pyrioides* | Tingidae | 21.8 | 31.7 | 3 | 3 | 0.40 | [182] |
| *Xylocoris flavipes* | Anthocoridae | 16 | 27.5 | 4 | 2.35 | 0.21 | [183] |
| *Zelus renardii* | Reduviidae | 34 | 30 | 3 | 15 | 30.17 | [172] |
| **Hemiptera, Sternorrhyncha** |  |  |  |  |  |  |  |
| *Acyrthosiphon kondoi* | Aphididae | 5.82 | 26.7 | 8 | 2.65 | 0.40 | [184] |
| *Acyrthosiphon pisum* | Aphididae | 7.3 | 23.1 | 5 | 3.4 | 0.70 | [185] |
| *Aleurocanthus woglumi* | Aleyrodidae | 54.6 | 34 | 6 | 1.5 | 0.11 | [186] |
| *Aleyrodes prolotella* | Aleyrodidae | 19 | 25 | 3 | 1.5 | 0.11 | [187] |
| *Aonidiella aurantii* | Diaspididae | 33.2 | 31.8 | 4 | 2.1 | 0.25 | [188] |
| *Aphis gossypii 6* | Aphididae | 4.6 | 30 | 6 | 1.6 | 0.13 | [189] |
| *Aphis nasturtii* | Aphididae | 5.7 | 30 | 7 | 1.3 | 0.08 | [190] |
| *Aphis pomi* | Aphididae | 4.7 | 28 | 4 | 1.75 | 0.15 | [191] |
| *Bemisia argentifolii* | Aleyrodidae | 13.6 | 30 | 5 | 1.5 | 0.11 | [192] |
| *Bemisia tabaci* | Aleyrodidae | 17.4 | 30.5 | 4 | 0.8 | 0.03 | [193] |
| *Cinara sp. nov.* | Aphididae | 9.21 | 25 | 4 | 1.9 | 0.19 | [194] |
| *Diaphorina citri* | Psyllidae | 14.06 | 28 | 5 | 3.5 | 0.86 | [195] |
| *Drepanosiphum acerinum* | Aphididae | 11.6 | 25 | 5 | 2.7 | 0.41 | [196] |
| *Drepanosiphum platanoids* | Aphididae | 10.2 | 25 | 5 | 3.7 | 0.86 | [196] |
| *Dysaphis plantaginea* | Aphididae | 11.6 | 19.5 | 3 | 2.3 | 0.29 | [197] |
| *Eriosoma lanigerum* | Aphididae | 11.65 | 30 | 6 | 1.6 | 0.13 | [198] |
| *Heteropsylla cubana* | Psyllidae | 11.6 | 30 | 3 | 2.5 | 0.38 | [199] |
| *Hyperomyzus lactucae* | Aphididae | 6.7 | 24 | 4 | 1.9 | 0.19 | [200] |
| *Lipaphis erysimi* | Aphididae | 5.2 | 30 | 4 | 1.4 | 0.09 | [201] |
| *Macrosiphum avenae* | Aphididae | 7.2 | 23 | 6 | 2.8 | 0.45 | [202] |
| *Macrosiphum euphorbiae* | Aphididae | 7.7 | 25 | 5 | 3.1 | 0.57 | [203] |
| *Metopolophium dirhodum* | Aphididae | 7.9 | 20.1 | 7 | 2.17 | 0.25 | [204] |
| *Phenacoccus manihoti* | Pseudococcidae | 27.3 | 27 | 4 | 3 | 0.59 | [205] |
| *Planococcus pacificus* | Pseudococcidae | 21 | 30 | 5 | 3.13 | 0.66 | [206] |
| *Psylla pyricola* | Psyllidae | 27 | 26.7 | 5 | 2.12 | 0.26 | [207] |
| *Rhopalosiphum padi* | Aphididae | 5.1 | 25 | 5 | 2 | 0.21 | [204] |
| *Rhopalosiphum rufiabdominalis* | Aphididae | 4.4 | 30 | 5 | 1.8 | 0.16 | [208] |
| *Sacchariococcus sacchari* | Pseudococcidae | 25.3 | 30 | 5 | 7 | 4.58 | [209] |
| *Sitobium avenae* | Aphididae | 6.9 | 25 | 5 | 3 | 0.53 | [210] |
| *Trialeurodes abutilonea* | Aleyrodidae | 20.7 | 35 | 3 | 1.5 | 0.11 | [211] |
| *Trialeurodes vaporariorum* | Aleyrodidae | 15.8 | 32 | 5 | 1.5 | 0.11 | [212] |
| **Hymenoptera** |  |  |  |  |  |  |  |
| *Acerophagus coccois* | Encyrtidae | 17 | 30 | 3 | 0.6 | 0.02 | [213] |
| *Amitus fuscipennis* | Platygastridae | 21.9 | 30 | 4 | 0.65 | 0.02 | [214] |
| *Anagyrus ananatis* | Encyrtidae | 15.4 | 31 | 5 | 1.73 | 0.16 | [215] |
| *Anagyrus pseudococci* | Encyrtidae | 10.5 | 33.8 | 6 | 1.13 | 0.06 | [216] |
| *Apanteles subandinus* | Braconidae | 11.9 | 32.2 | 6 | 3.9 | 0.96 | [217] |
| *Aphelinus asychis* | Aphelinidae | 11.7 | 26 | 4 | 1 | 0.05 | [218] |
| *Aphelinus gossypii* | Aphelinidae | 14 | 30 | 6 | 0. | 0.01 | [219] |
| *Aphelinus semiflavus* | Aphelinidae | 10.4 | 29.4 | 4 | 0.6 | 0.02 | [220] |
| *Aphelinus spiraecolae* | Aphelinidae | 13.3 | 30 | 6 | 1.04 | 0.05 | [219] |
| *Aphidius colemani* | Braconidae | 9.8 | 25 | 5 | 2.5 | 0.35 | [221] |
| *Aphidius matricariae* | Braconidae | 11.6 | 25 | 5 | 2.5 | 0.35 | [221] |
| *Aphytis chrysomphali* | Aphelinidae | 13.3 | 30 | 3 | 0.75 | 0.03 | [222] |
| *Aphytis melanis* | Aphelinidae | 13.1 | 30 | 3 | 0.86 | 0.04 | [222] |
| *Bathyplectes curculionis* | Ichneumonidae | 7.1 | 26.7 | 4 | 3 | 0.53 | [52] |
| *Bracon vulgaris* | Braconidae | 8.63 | 35 | 8 | 8 | 5.06 | [223] |
| *Catolaccus grandis* | Pteromalidae | 11.8 | 33 | 6 | 4.25 | 1.16 | [224] |
| *Copidosoma koehleri* | Encyrtidae | 25.3 | 32.6 | 5 | 1.5 | 0.12 | [225] |
| *Cotesia flavipes* | Braconidae | 15.2 | 31 | 4 | 2 | 0.22 | [226] |
| *Cotesia sesamiae* | Braconidae | 15.1 | 31 | 4 | 2 | 0.22 | [226] |
| *Dendrosoter sulcatus* | Braconidae | 11.7 | 30 | 4 | 3.15 | 0.59 | [227] |
| *Diadegma anurum* | Ichneumonidae | 12.69 | 30 | 4 | 3.9 | 0.96 | [228] |
| *Dibrachys boarmiae* | Pteromalidae | 12.6 | 32.5 | 9 | 2.48 | 0.35 | [229] |
| *Diglyphus isaea* | Eulophidae | 6.45 | 35 | 5 | 2.5 | 0.35 | [230] |
| *Dinotiscus dendroctoni* | Pteromalidae | 16.2 | 30 | 4 | 2.7 | 0.42 | [227] |
| *Edovum puttleri* | Eulophidae | 11.2 | 29.4 | 7 | 1.5 | 0.12 | [231] |
| *Encarsia citrina* | Aphelinidae | 22.8 | 27.5 | 4 | 0.5 | 0.01 | [232] |
| *Encarsia formosa* | Aphelinidae | 13.2 | 30 | 3 | 0.6 | 0.02 | [233] |
| *Encarsia tricolor* | Aphelinidae | 14.5 | 28.7 | 9 | 1 | 0.05 | [234] |
| *Ephedrus californicus* | Braconidae | 12.3 | 26.4 | 4 | 2.25 | 0.28 | [235] |
| *Eretmocerus eremicus* | Aphelinidae | 16.5 | 30.9 | 3 | 1 | 0.05 | [236] |
| *Goniozus legneri* | Bethylidae | 8.1 | 35 | 9 | 3.25 | 0.63 | [237] |
| *Gronotoma micromorpha* | Figitidae | 16.2 | 30 | 5 | 1.2 | 0.07 | [238] |
| *Heydenia unica* | Pteromalidae | 18.2 | 30 | 4 | 3.5 | 0.75 | [227] |
| *Leptomastidea abnormis* | Encyrtidae | 19.4 | 30.3 | 5 | 1.13 | 0.06 | [239] |
| *Leptomastix dactylopii* | Encyrtidae | 12.3 | 35.7 | 6 | 3 | 0.53 | [239] |
| *Lysiphlebus testaceipes* | Braconidae | 9.5 | 30 | 6 | 2 | 0.22 | [219] |
| *Macrocentrus grandii* | Braconidae | 26.3 | 30 | 4 | 4.75 | 1.50 | [240] |
| *Macrocentrus iridescens* | Braconidae | 36.5 | 25.8 | 6 | 7 | 3.70 | [241] |
| *Muscidifurax raptor* | Pteromalidae | 12.6 | 31.1 | 5 | 1.5 | 0.12 | [242] |
| *Muscidifurax raptorellus* | Pteromalidae | 12.45 | 30.9 | 5 | 1.4 | 0.10 | [243] |
| *Muscidifurax zaraptor* | Pteromalidae | 13.25 | 29.8 | 5 | 2.5 | 0.35 | [244] |
| *Nasonia vitripennis* | Pteromalidae | 11.3 | 30 | 4 | 2.5 | 0.35 | [245] |
| *Oomyzus sokolowskii* | Eulophidae | 12.7 | 30 | 3 | 1.5 | 0.12 | [246] |
| *Philanthus triangulum* | Crabronidae | 20.2 | 30 | 3 | 15 | 22.79 | [247] |
| *Praon palitans* | Braconidae | 14.2 | 23.9 | 3 | 2.1 | 0.24 | [220] |
| *Praon pequodorum* | Braconidae | 10.5 | 25.9 | 4 | 2.25 | 0.28 | [248] |
| *Prorops nasuta* | Bethylidae | 16 | 30 | 4 | 2.5 | 0.35 | [249] |
| *Spalangia cameroni* | Pteromalidae | 22.5 | 30 | 4 | 2.85 | 0.47 | [250] |
| *Spalangia endius* | Pteromalidae | 15.4 | 35 | 4 | 2.45 | 0.34 | [250] |
| *Spalangia gemina* | Pteromalidae | 18.9 | 30 | 6 | 3.35 | 0.68 | [251] |
| *Spathius pallidus* | Braconidae | 12.8 | 30 | 4 | 3.5 | 0.75 | [227] |
| *Telenomus chrysopae* | Scelionidae | 8.7 | 26.7 | 5 | 1.7 | 0.15 | [252] |
| *Telenomus reynoldsi* | Scelionidae | 9.4 | 33 | 7 | 0.78 | 0.03 | [253] |
| *Telenomus utahensis* | Scelionidae | 6.3 | 35 | 5 | 0.8 | 0.03 | [254] |
| *Trichogramma evanescens* | Trichogrammatidae | 8.3 | 34 | 4 | 0.35 | 0.01 | [255] |
| *Trichogramma minutum* | Trichogrammatidae | 9.4 | 27 | 3 | 0.5 | 0.01 | [256] |
| *Trichogramma ostriniae* | Trichogrammatidae | 7 | 30.3 | 6 | 0.4 | 0.01 | [257] |
| *Trichogramma pretiosum* | Trichogrammatidae | 7.7 | 32.5 | 5 | 0.32 | 0.004 | [258] |
| *Trichomalopsis sarcophagae* | Pteromalidae | 9.5 | 33 | 5 | 2 | 0.22 | [259] |
| *Trioxys utilis* | Braconidae | 9.3 | 29.4 | 4 | 2.25 | 0.28 | [220] |
| *Trissolcus brochymenae* | Scelionidae | 9 | 32 | 5 | 1.2 | 0.07 | [260] |
| *Trissolcus oenone* | Scelionidae | 9.7 | 30 | 8 | 1.8 | 0.17 | [261] |
| *Uscana lariophaga* | Trichogrammatidae | 6.8 | 35 | 6 | 0.45 | 0.01 | [262] |
| **Lepidoptera** |  |  |  |  |  |  |  |
| *Anarsia lineatella* | Gelechiidae | 20.6 | 30 | 5 | 5.5 | 1.50 | [263] |
| *Calophasia lunula* | Noctuidae | 24.9 | 30 | 5 | 13 | 18.28 | [264] |
| *Cryptophlebia illepida* | Tortricidae | 33.3 | 24.3 | 3 | 9 | 6.26 | [265] |
| *Cydia pomonella* | Tortricidae | 31.48 | 30 | 6 | 10 | 8.51 | [266] |
| *Diatraea lineolata* | Crambidae | 39.4 | 31 | 4 | 12 | 14.48 | [267] |
| *Elasmopalpus lignosellus* | Pyralidae | 22.8 | 33 | 9 | 18.5 | 51.31 | [268] |
| *Ephestia kuehniella* | Pyralidae | 40.6 | 27.5 | 4 | 9.5 | 7.33 | [269] |
| *Episimus utilis* | Tortricidae | 31 | 30 | 4 | 5.5 | 1.50 | [270] |
| *Euzopherodes vapidella* | Pyralidae | 22 | 33 | 4 | 6.95 | 2.95 | [271] |
| *Heliothis zea* | Noctuidae | 21.5 | 32 | 7 | 19 | 55.47 | [272] |
| *Hyphantria cunea* | Arctiidae | 39.7 | 27 | 6 | 13 | 18.28 | [273] |
| *Mamestra configurata* | Noctuidae | 39.1 | 28 | 6 | 20 | 64.47 | [274] |
| *Maruca vitrata* | Crambidae | 17.9 | 29.3 | 5 | 11 | 11.23 | [275] |
| *Mythimna convecta* | Noctuidae | 28.3 | 33 | 5 | 30 | 211.9 | [276] |
| *Pectinophora gossypiella* | Gelechiidae | 25.3 | 32 | 3 | 8.5 | 8.5 | [277] |
| *Phthorimaea operculella* | Gelechiidae | 16 | 32.4 | 7 | 10 | 8.51 | [225] |
| *Plutella xylostella* | Plutellidae | 12.8 | 30.5 | 5 | 6 | 1.93 | [278] |
| *Sitotroga cerealella* | Gelechiidae | 37.44 | 30 | 4 | 7.5 | 3.68 | [279] |
| **Neuroptera** |  |  |  |  |  |  |  |
| *Anomalochrysa frater* | Chrysopidae | 25.8 | 28 | 5 | 9.6 | 3.19 | [280] |
| *Anomalochrysa hepatica* | Chrysopidae | 26.5 | 23.4 | 4 | 12.2 | 5.37 | [281] |
| *Chrysopa carnea* | Chrysopidae | 15.5 | 35 | 4 | 7.66 | 1.98 | [282] |
| *Chrysopa harrisii* | Chrysopidae | 23.5 | 26.7 | 4 | 14 | 7.29 | [283] |
| *Chrysopa oculata* | Chrysopidae | 40.5 | 21.1 | 3 | 12 | 5.18 | [284] |
| *Micromus tasmaniae* | Hemerobiidae | 17.5 | 28 | 3 | 8.8 | 2.65 | [165] |
| **Odonata** |  |  |  |  |  |  |  |
| *Argia vivida* | Coenagrionidae | 57.1 | 25 | 3 | 33.5 | 192.13 | [285] |
| *Coenagrion puella* | Coenagrionidae | 196.4 | 24 | 5 | 34 | 200.23 | [286] |
| **Orthoptera** |  |  |  |  |  |  |  |
| *Acheta domesticus* | Gryllidae | 35 | 35 | 5 | 18 | 52.70 | [287] |
| *Chorthippus brunneus* | Acrididae | 19.9 | 40 | 4 | 19 | 60.46 | [288] |
| *Chortoicetes terminifera* | Acrididae | 28.2 | 39 | 6 | 27.5 | 154.9 | [289] |
| *Gryllus assimilis* | Gryllidae | 77.8 | 30 | 3 | 22 | 87.79 | [290] |
| *Gryllus bimaculatus* | Gryllidae | 60.8 | 30 | 3 | 27 | 147.83 | [290] |
| *Melanoplus femurrubrum* | Acrididae | 29.3 | 38 | 4 | 22.8 | 96.14 | [291] |
| *Melanoplus mexicanus* | Acrididae | 59 | 32 | 4 | 24.5 | 115.44 | [292] |
| *Melanoplus sanguinipes* | Acrididae | 15.8 | 39 | 8 | 24.5 | 115.44 | [293] |
| *Taeniopoda eques* | Acrididae | 27.1 | 35 | 3 | 59 | 1082.47 | [294] |
| *Teleogryllus commodus* | Gryllidae | 84.4 | 30 | 3 | 30 | 193.30 | [290] |
| **Phasmatodea** |  |  |  |  |  |  |  |
| *Baculum elongatus* | Phasmatidae | 42.8 | 28 | 4 | 79 | 1499.24 | [295] |
| **Psocodea** |  |  |  |  |  |  |  |
| *Liposcelis bostrychophila* | Liposcelididae | 18.1 | 32.5 | 7 | 1.1 | 0.05 | [296] |
| *Liposcelis paeta* | Liposcelididae | 11.5 | 37.5 | 7 | 1.17 | 0.06 | [297] |
| *Liposcelis rufa* | Liposcelididae | 19.55 | 37.5 | 8 | 1.35 | 0.07 | [298] |
| **Siphonaptera** |  |  |  |  |  |  |  |
| *Ctenocephalides felis* | Pulicidae | 16.8 | 32 | 4 | 2 | 0.15 | [299] |
| *Nosopsyllus laeviceps kuzenkovi* | Ceratophyllidae | 20.68 | 26 | 5 | 2.5 | 0.26 | [300] |
| *Spilopsyllus cuniculi* | Pulicidae | 15.9 | 30 | 4 | 1 | 0.03 | [301] |
| *Xenopsylla astia* | Pulicidae | 7.6 | 35 | 7 | 1.87 | 0.13 | [302] |
| *Xenopsylla brasiliensis* | Pulicidae | 20.4 | 31.9 | 5 | 1.64 | 0.09 | [302] |
| *Xenopsylla cheopis* | Pulicidae | 18.5 | 31.9 | 7 | 1.85 | 0.13 | [302] |
| **Thysanoptera** |  |  |  |  |  |  |  |
| *Frankliniella fusca* | Thripidae | 11.5 | 35 | 4 | 1.3 | 0.01 | [303] |
| *Frankliniella occidentalis* | Thripidae | 10.2 | 31 | 6 | 1.2 | 0.01 | [304] |
| *Gynaikothrips ficorum* | Phlaeothripidae | 16 | 30 | 4 | 3.1 | 0.13 | [305] |
| *Megalurothrips sjostedti* | Thripidae | 9.7 | 30 | 4 | 2 | 0.04 | [306] |
| *Scirtothrips perseae* | Thripidae | 16.35 | 30 | 5 | 0.9 | 0.01 | [307] |
| *Scolothrips sexmaculatus* | Thripidae | 11 | 30 | 4 | 2.5 | 0.07 | [308] |
| *Thrips major* | Thripidae | 13.4 | 28 | 5 | 1.4 | 0.02 | [309] |
| *Thrips obscuratus* | Thripidae | 10 | 27.5 | 7 | 0.9 | 0.01 | [310] |
| *Thrips palmi* | Thripidae | 10.9 | 32 | 3 | 0.9 | 0.01 | [311] |
| *Thrips tabaci* | Thripidae | 10.63 | 30 | 5 | 1.3 | 0.01 | [312] |

**Table S1 References**

1. Benson EP, Zungoli PA, Smith LM (1994) Comparison of developmental rates of two separate populations of *Periplaneta fuliginosa* (Dictyoptera: Blattidae) and equations describing development, preoviposition, and oviposition. Environ Entomol 23: 979–986.

2. Tsai T-J, Chi H (2007) Temperature-dependent demography of *Supella longipalpa* (Blattodea: Blattellidae). J Med Entomol 44: 772–778. doi:10.1603/0022-2585(2007)44[772:TDOSLB]2.0.CO;2.

3. Obrycki JJ, Tauber MJ (1981) Phenology of three Coccinellid species: thermal requirements for development. Ann Entomol Soc Am 74: 31–36.

4. Jacob TA (1996) The effect of constant temperature and humidity on the development, longevity and productivity of *Ahasverus advena* (Waltl.)(Coleoptera: Silvanidae). J Stored Prod Res 32: 115–121.

5. Rueda LM, Axtell RC (1996) Temperature-dependent development and survival of the lesser mealworm, *Alphitobius diaperinus*. Med Vet Entomol 10: 80–86.

6. Saska P, Honek A (2003) Temperature and development of central European species of *Amara* (Coleoptera: Carabidae). Eur J Entomol 100: 509–516.

7. Keena MA, Moore PM (2010) Effects of temperature on *Anoplophora glabripennis* (Coleoptera: Cerambycidae) larvae and pupae. Environ Entomol 39: 1323–1335. doi:10.1603/EN09369.

8. Toapanta MA, Schuster DJ, Stansly PA (2005) Development and life history of *Anthonomus eugenii* (Coleoptera: Curculionidae) at constant temperatures. Environ Entomol 34: 999–1008. doi:10.1603/0046-225X(2005)034[0999:DALHOA]2.0.CO;2.

9. Fye RE, Patana R, McAda WC (1969) Developmental periods for boll weevil reared at several constant and fluctuating temperatures. J Econ Entomol 62: 377–380.

10. Clarke RG, Howitt AJ (1975) Development of the strawberry weevil under laboratory and field conditions. Ann Entomol Soc Am 68: 715–718.

11. Marco V, Taberner A, Castañera P (1997) Development and survival of immature *Aubeonymus mariaefranciscae* (Coleoptera: Curculionidae) at constant temperatures. Ann Entomol Soc Am 90: 169–176.

12. Godfrey KE, Anderson LWJ (1994) Developmental rates of *Bagous affinis* (Coleoptera: Curculionidae) at constant temperatures. Fla Entomol 77: 516–519.

13. Sherrod DW, White CE, Eastman CE (1982) Temperature-related development of the imported crucifer weevil, *Baris lepidii* (Coleoptera: curculionidae), in the laboratory and field. Environ Entomol 11: 897–900.

14. Giga DP, Smith RH (1983) Comparative life history studies of four *Callosobruchus* species infesting cowpeas with special reference to *Callosobruchus rhodesianus* (Pic) (Coleoptera: Bruchidae). J Stored Prod Res 19: 189–198. doi:10.1016/0022-474X(83)90007-3.

15. Lale NES, Vidal S (2003) Effect of constant temperature and humidity on oviposition and development of *Callosobruchus maculatus* (F.) and *Callosobruchus subinnotatus* (Pic) on bambara groundnut, *Vigna subterranea* (L.) Verdcourt. J Stored Prod Res 39: 459–470.

16. LaMana ML, Miller JC (1998) Temperature-dependent development in an Oregon population of *Harmonia axyridis* (Coleoptera: Coccinellidae). Environ Entomol 27: 1001–1005.

17. Porter J (1986) Some studies on the life history and oviposition of *Carpophilus dimidiatus* (F.)(Coleoptera: Nitidulidae) at various temperatures and humidities. J Stored Prod Res 22: 135–139.

18. Ward RH, Pienkowski RL (1978) Biology of *Cassida rubiginosa* a thistle-feeding shield beetle. Ann Entomol Soc Am 71: 585–591.

19. Nava DE, Parra JRP (2003) Biology of *Cerotoma arcuatus* (Coleoptera: Chrysomelidae) and field validation of a laboratory model for temperature requirements. J Econ Entomol 96: 609–614. doi:10.1603/0022-0493-96.3.609.

20. Ponsonby DJ, Copland MJW (1996) Effect of temperature on development and immature survival in the scale insect predator, *Chilocorus nigritus* (F.) (Coleoptera: Coccinellidae). Biocontrol Sci Technol 6: 101–110. doi:10.1080/09583159650039566.

21. Xu P, Zheng Y-L, Lv X-Z, Yao S-T, Chen X-X (2009) Biological characteristics of *Cionus latefasciatus* (Coleoptera: Curculionidae) and effects of temperature on its growth and development. J Econ Entomol 102: 1039–1043. doi:10.1603/029.102.0323.

22. McMullen RD (1967) The effects of photoperiod, temperature, and food supply on rate of development and diapause in *Coccinella novemnotata*. Can Entomol 99: 578–586. doi:10.4039/Ent99578-6.

23. Katsarou I, Margaritopoulos JT, Tsitsipis JA, Perdikis DC, Zarpas KD (2005) Effect of temperature on development, growth and feeding of *Coccinella septempunctata* and *Hippodamia convergens* reared on the tobacco aphid, *Myzus persicae nicotianae*. BioControl 50: 565–588. doi:10.1007/s10526-004-2838-1.

24. Obrycki JJ, Tauber MJ (1981) Phenology of three Coccinellid species: thermal requirements for development. Ann Entomol Soc Am 74: 31–36.

25. Miller JC, LaMana ML (1995) Assessment of temperature-dependent development in the general population and among isofemale lines of *Coccinella trifasciata* (Col.: Coccinellidae). BioControl 40: 183–192.

26. Wright EJ, Laing JE (1978) Effects of temperature on development, adult longevity and fecundity of *Coleomegilla maculata* Lengi and its parasite, *Perilitus coccinellae*. Proc Entomol Soc Ont 109: 33–47.

27. Butler Jr. GD, Wardecker AL (1973) *Collops vittatus* (Coleoptera: Malachiidae): Development at constant temperatures. Ann Entomol Soc Am 66: 1168–1170.

28. Lan Z, Scherm H, Horton DL (2004) Temperature-dependent development and prediction of emergence of the summer generation of plum *Curculio* (coleoptera: curculionidae) in the Southeastern United States. Environ Entomol 33: 174–181. doi:10.1603/0046-225X-33.2.174.

29. Taylor RG, Harcourt DG (1978) Effects of temperature on developmental rate of the immature stages of *Crioceris asparagi* (Coleoptera: Chrysomelidae). Can Entomol 110: 57–62. doi:10.4039/Ent11057-1.

30. Smith LB (1965) The intrinsic rate of natural increase of *Cryptolestes ferrugineus* (Stephens)(Coleoptera, Cucujidae). J Stored Prod Res 1: 35–49.

31. Currie JE (1967) Some effects of temperature and humidity on the rates of development, mortality and oviposition of *Crvptolestes pusillus* (Schönherr)(Coleoptera, Cucujidae). J Stored Prod Res 3: 97–108.

32. Lefkovitch LP (1962) The biology of *Cryptolestes turcicus* (Grouvelle) (Coleoptera: Cucujidae), a pest of stored and processed cereals. Proc Zool Soc Lond 138: 23–35. doi:10.1111/j.1469-7998.1962.tb05685.x.

33. Mullen MA (1981) Sweetpotato Weevil, *Cylas formicarius elegantulus* (Summers): Development, fecundity, and longevity. Ann Entomol Soc Am 74: 478–481.

34. Nteletsana L, Schoeman AS, McGeoch MA (2001) Temperature effects on development and survival of the sweetpotato weevil, *Cylas puncticollis* Boheman (Coleoptera: Apionidae). Afr Entomol 9: 49–57.

35. Bentz B, Logan J, Amman G (1991) Temperature-dependent development of the mountain pine beetle (Coleoptera : Scolytidae) and simulation of its phenology. Can Entomol 123: 1083–1094.

36. Coombs CW (1981) The development, fecundity and longevity of *Dermestes ater* Degeer (Coleoptera: Dermestidae). J Stored Prod Res 17: 31–36.

37. Amos T (1968) Some laboratory observations on the rates of development, mortality and oviposition of *Dermestes frischii* (Kug.)(Col., Dermestidae). J Stored Prod Res 4: 103–117.

38. Coombs CW (1979) The effect of temperature and humidity upon the development and fecundity of *Dermestes haemorrhoidalis* Küster and *Dermestes peruvianus* Laporte de Castelnau (Coleoptera: Dermestidae). J Stored Prod Res 15: 43–52.

39. Richardson MS, Goff ML (2001) Effects of temperature and intraspecific interaction on the development of *Dermestes maculatus* (Coleoptera: Dermestidae). J Med Entomol 38: 347–351. doi:10.1603/0022-2585-38.3.347.

40. Chong JH, Oetting RD, Osborne LS (2005) Development of *Diomus austrinus* Gordon (Coleoptera: Coccinellidae) on two mealybug prey species at five constant temperatures. Biol Control 33: 39–48.

41. Herrera AM, Dahlsten DD, Tomic-Carruthers N, Carruthers RI (2005) Estimating temperature-dependent developmental rates of *Diorhabda elongata* (Coleoptera: Chrysomelidae), a biological control agent of saltcedar (*Tamarix* spp.). Environ Entomol 34: 775–784. doi:10.1603/0046-225X-34.4.775.

42. Fan Y, Groden E, Drummond FA (1992) Temperature-dependent development of Mexican bean beetle (Coleoptera: Coccinellidae) under constant and variable temperatures. J Econ Entomol 85: 1762–1770.

43. Mazzei KC, Newman RM, Loos A, Ragsdale DW (1999) Developmental rates of the native milfoil weevil, *Euhrychiopsis lecontei*, and damage to eurasian watermilfoil at constant temperatures. Biol Control 16: 139–143. doi:10.1006/bcon.1999.0739.

44. McAvoy TJ, Kok LT (2004) Temperature dependent development and survival of two sympatric species, *Galerucella calmariensis* and *G. pusilla*, on purple loosestrife. Biocontrol 49: 467–480.

45. Honek A, Jarosik V, Martinkova Z (2003) Effect of temperature on development and reproduction in *Gastrophysa viridula* (Coleoptera: Chrysomelidae). Eur J Entomol 100: 295–300.

46. Mussen EC, Chiang HC (1974) Development of the picnic beetle, *Glischrochilus quadrisignatus* (Say), at various temperatures. Environ Entomol 3: 1032–1034.

47. Ntifo SEA, Nowosielski-Slepowron BJA (1973) Developmental period and mortality of *Gnathocerus maxillosus* (F.)(Coleoptera, Tenebrionidae) under various conditions of temperature and humidity. J Stored Prod Res 9: 51–59.

48. Santolamazza-Carbone S, Rodriguez-Illamola A, Cordero AR (2006) Thermal requirements and phenology of the *Eucalyptus* snout beetle *Gonipterus scutellatus* Gyllenhal. J Appl Entomol 130: 368–376. doi:10.1111/j.1439-0418.2006.01073.x.

49. Orr CJ, Obrycki JJ (1990) Thermal and dietary requirements for development of *Hippodamia parenthesis* (Coleoptera: Coccinellidae). Environ Entomol 19: 1523–1527.

50. Michels Jr. GJ, Behle RW (1991) Effects of two prey species on the development of *Hippodamia sinuate* (Coleoptera: Coccinellidae) larvae at constant temperatures. J Econ Entomol 84: 1480–1484.

51. Salom SM, Stephen FM, Thompson LC (1987) Development rates and a temperature-dependent model of pales weevil, *Hylobius pales* (Herbst), development. Environ Entomol 16: 956–962.

52. Butler Jr. GD, Ritchie Jr. PL (1967) The life cycle of *Hypera brunneipennis* and a parasite, *Bathyplectes curculionis*, in relation to temperature. J Econ Entomol 60: 1239–1241.

53. Chan W-P, Ellsbury MM, Baker GT (1990) Effects of temperature on preimaginal development of *Hypera meles* (Coleoptera: Curculionidae). Ann Entomol Soc Am 83: 1116–1124.

54. Guppy JC, Mukerji MK (1974) Effects of temperature on developmental rate of the immature stages of the alfalfa weevil, *Hypera postica* (Coleoptera: Curculionidae). Can Entomol 106: 93–100. doi:10.4039/Ent10693-1.

55. Dreyer BS, Neuenschwander P, Bouyjou B, Baumgärtner J, Dorn S (1997) The influence of temperature on the life table of *Hyperaspis notata*. Entomol Exp Appl 84: 85–92.

56. Wagner TL, Hennier PB, Flamm RO, Coulson RN (1988) Development and mortality of *Ips avulsus* (Coleoptera: Scolytidae) at constant temperatures. Environ Entomol 17: 181–191.

57. Wagner TL, Fargo WS, Flamm RO, Coulson RN, Pulley PE (1987) Development and mortality of *Ips calligraphus* (Coleoptera: Scolytidae) at constant temperatures. Environ Entomol 16: 484–496.

58. Berryman AA, Stark RW (1962) Studies on the effects of temperature on the development of *Ips confusus* using radiographic techniques. Ecology 43: 722–726.

59. Wermelinger B, Seifert M (1999) Temperature‐dependent reproduction of the spruce bark beetle *Ips typographus*, and analysis of the potential population growth. Ecol Entomol 24: 103–110. doi:10.1046/j.1365-2311.1999.00175.x.

60. Zilahi-Balogh GMG, Salom SM, Kok LT (2003) Temperature-dependent development of the specialist predator *Laricobius nigrinus* (Coleoptera: Derodontidae). Environ Entomol 32: 1322–1328. doi:10.1603/0046-225X-32.6.1322.

61. Nowosielski-Slepowron BJA, Aryeetey EA (1980) Developmental biology of field and laboratory populations of *Latheticus oryzae* Waterhouse (Coleoptera, Tenebrionidae) under various conditions of temperature and humidity. J Stored Prod Res 16: 55–66.

62. Logan PA, Casagrande RA, Faubert HH, Drummond FA (1985) Temperature-dependent development and feeding of immature Colorado potato beetles, *Leptinotarsa decemlineata* (Say) (Coleoptera: Chrysomelidae). Environ Entomol 14: 275–283.

63. Brown HD (1972) On the biology of *Lioadalia flavomaculara* (Deg.) (Col., Coccineffidae), a predator of the wheat aphid (*Schizaphis graminum* (rond.)) in South Africa. Bull Entomol Res 61: 673–679. doi:10.1017/S0007485300047507.

64. Simonet DE, Davenport BL (1981) Temperature requirements for development and oviposition of the carrot weevil [*Listronotus oregonensis*]. Ann Entomol Soc Am 74: 312–315.

65. Pershing JC, Linit MJ (1986) Development and seasonal occurrence of *Monochamus carolinensis* (Coleoptera: Cerambycidae) in Missouri. Environ Entomol 15: 251–253.

66. Ren S-X, Stansly PA, Liu TX (2002) Life history of the whitefly predator *Nephaspis oculatus* (Coleoptera: Coccinellidae) at six constant temperatures. Biol Control 23: 262–268.

67. Jacob TA (1981) Observations on the biology of *Oryzaephilus acuminatus* Halstead with comparative notes on the common species of *Oryzaephilus* (Coleoptera; Silvanidae). J Stored Prod Res 17: 17–23.

68. Beckett SJ, Evans DE (1994) The demography of *Oryzaephilus surinamensis* (L.) (Coleoptera: Silvanidae) on kibbled wheat. J Stored Prod Res 30: 121–137.

69. Stenseth C (1979) Effects of temperature on development of *Otiorrhynchus sulcatus* (Coleoptera: Curculionidae). Ann Appl Biol 91: 179–185. doi:10.1111/j.1744-7348.1979.tb06488.x.

70. Guppy JC, Harcourt DG (1978) Effects of temperature on development of the immature stages of the cereal leaf beetle, *Oulema melanopus* (Coleoptera: Chrysomelidae). Can Entomol 110: 257–263.

71. Halstead DGH (1967) Biological studies on species of *Palorus* and *Coelopalorus* with comparative notes on *Tribolium* and *Latheticus* (Coleoptera: Tenebrionidae). J Stored Prod Res 2: 273–313.

72. Ludwig D (1928) The effects of temperature on the development of an insect (*Popillia japonica* Newman). Physiol Zool 1: 358–389.

73. Omkar, Pervez A (2004) Temperature‐dependent development and immature survival of an aphidophagous ladybeetle, *Propylea dissecta* (Mulsant). J Appl Entomol 128: 510–514. doi:10.1111/j.1439-0418.2004.00881.x.

74. Bell RJ, Watters FL (1982) Environmental factors influencing the development and rate of increase of *Prostephanus truncatus* (Horn) (Coleoptera: Bostrichidae) on stored maize. J Stored Prod Res 18: 131–142. doi:10.1016/0022-474X(82)90013-3.

75. Allsopp PG (1981) Development, longevity and fecundity of the false wireworms *Pterohelaeus darlingensis* and *P. alternatus* (Coleoptera: Tenebrionidae). I. Effect of constant temperature. Aust J Zool 29: 605–619.

76. King JE, Price RG, Young JH, Willson LJ, Pinkston KN (1985) Influence of temperature on development and survival of the immature stages of the elm leaf beetle, *Pyrrhalta luteola* (Muller) (Coleoptera: Chrysomelidae). Environ Entomol 14: 272–274.

77. Birch LC (1953) Experimental background to the study of the distribution and abundance of insects. I. The influence of temperature, moisture, and food on the innate capacity for increase of three grain beetles. Ecology 34: 698–711.

78. Stathas GJ (2000) The effect of temperature on the development of the predator *Rhyzobius lophanthae* and its phenology in Greece. BioControl 45: 439–451.

79. Grafton-Cardwell EE, Gu P, Montez GH (2005) Effects of temperature on development of vedalia beetle, *Rodolia cardinalis* (Mulsant). Biol Control 32: 473–478.

80. Ryoo MI, Cho K-J (1988) A model for the temperature-dependent developmental rate of *Sitophilus oryzae* L. (Coleoptera: Curculionidae) on rice. J Stored Prod Res 24: 79–82. doi:10.1016/0022-474X(88)90034-3.

81. Lefkovitch LP (1967) A laboratory study of *Stegobium paniceum* (L.)(Coleoptera: Anobiidae). J Stored Prod Res 3: 235–249.

82. Roy M, Brodeur J, Cloutier C (2002) Relationship between temperature and developmental rate of *Stethorus punctillum*(Coleoptera: Coccinellidae) and its prey *Tetranychus mcdanieli* (Acarina: Tetranychidae). Environ Entomol 31: 177–187.

83. Howe RW (1962) Observations on the rate of growth and disruption of moulting in the larvae and pupae of *Tribolium castaneum* (Herbst)(Coleoptera, Tenebrionidae) at sub-threshold temperatures. Entomol Exp Appl 5: 211–222.

84. Howe RW (1960) The effects of temperature and humidity on the rate of development and the mortality of *Tribolium confusum* Duval (Coleoptera, Tenebrionidae). Ann Appl Biol 48: 363–376. doi:10.1111/j.1744-7348.1960.tb03532.x.

85. Imura O, Nakakita H (1984) The effect of temperature and relative humidity on the development of *Tribolium freemani* Hinton (Coleoptera: Tenebrionidae). J Stored Prod Res 20: 87–95.

86. Howe RW (1962) The effect of temperature and relative humidity on the rate of development and the mortality of *Tribolium madens* (Charp.) (Coleoptera, Tenebrionidae). Ann Appl Biol 50: 649–660. doi:10.1111/j.1744-7348.1962.tb06065.x.

87. Burges HD, Cammell ME (1964) Effect of temperature and humidity on *Trogoderma anthrenoides* (Sharp)(Coleoptera, Dermestidae) and comparisons with related species. Bull Entomol Res 55: 313–325. doi:10.1017/S0007485300049476.

88. Archer TL, Strong RG (1975) Comparative Studies on the Biologies of Six Species of *Trogoderma*: *T. glabrum*. Ann Entomol Soc Am 68: 105–114.

89. Partida GJ, Strong RG (1975) Comparative studies on the biologies of six species of *Trogoderma*: *T. variabile*. Ann Entomol Soc Am 68: 115–125.

90. Hadaway AB (1956) The biology of the dermestid beetles, *Trogoderma granarium* Everts and *Trogoderma versicolor* (Creutz.). Bull Entomol Res 46: 781–796. doi:10.1017/S0007485300037044.

91. Jacob TA (1988) The effect of temperature and humidity on the developmental period and mortality of *Typhaea stercorea* (L.)(Coleoptera: Mycetophagidae). J Stored Prod Res 24: 221–224.

92. Stam EM, Leemkule MA, Ernsting G (1996) Trade-offs in the life history and energy budget of the parthenogenetic collembolan *Folsomia candida* (Willem). Oecologia 107: 283–292.

93. Birkemoe T, Leinaas H (2000) Effects of temperature on the development of an arctic Collembola (*Hypogastrura tullbergi*). Funct Ecol 14: 693–700.

94. Choi WI, Ryoo MI, Kim JG (2002) Biology of *Paronychiurus kimi* (Collembola: Onychiuridae) under the influence of temperature, humidity and nutrition. Pedobiologia 46: 548–557.

95. Snider RM (1974) The life cycle relative to temperature of *Protaphorura armatus* (Tullberg)(Collembola: Onychiuridae), a parthenogenetic species. Gt Lakes Entomol 7: 15.

96. Aroga R, Coderre D (2001) Effects of temperature on the development and fecundity of *Diaperasticus erythrocephala* Olivier (Dermaptera: Forficulidae). Insect Sci Its Appl 21: 161–167.

97. Lemos WP, Medeiros RS, Ramalho FS (1998) Influência da temperatura no desenvolvimento de *Euborellia annulipes* (Lucas)(Dermaptera: Anisolabididae), predador do Bicudo-do-Algodoeiro. An Soc Entomológica Bras 27: 67–76.

98. Simpson GB (1993) Effects of temperature on the development, longevity and fecundity of *Nala lividipes* (Dufour)(Dermaptera: Labiduridae). Aust J Entomol 32: 265–272.

99. Delatte H, Gimonneau G, Triboire A, Fontenille D (2009) Influence of temperature on immature development, survival, longevity, fecundity, and gonotrophic cycles of *Aedes albopictus*, vector of chikungunya and dengue in the Indian ocean. J Med Entomol 46: 33–41. doi:10.1603/033.046.0105.

100. Tauthong P, Brust TA (1977) The effect of temperature on the development and survival of two populations of *Aedes campestris* Dyar and Knab (Diptera: Culicidae). Can J Zool 55: 135–137.

101. Bayoh MN, Lindsay SW (2003) Effect of temperature on the development of the aquatic stages of *Anopheles gambiae* sensu stricto (Diptera: Culicidae). Bull Entomol Res 93: 375–382.

102. Huffaker CB (1944) The temperature relations of the immature stages of the malarial mosquito, *Anopheles quadrimaculatus* Say, with a comparison of the developmental power of constant and variable temperatures in insect metabolism. Ann Entomol Soc Am 37: 1–27.

103. Havelka J (1980) Effect of temperature on the developmental rate of preimaginal stages of *Aphidoletes aphidimyza* (Diptera, Cecidomyiidae). Entomol Exp Appl 27: 83–90.

104. Delobel A, Unnithan G (1983) Influence des températures constantes sur les caractéristiques des populations d’*Atherigona soccata* (Diptères Muscidae). Acta Oecologica Oecologia Appl 4: 351–368.

105. Yang P, Carey JR, Dowell RV (1994) Temperature influences on the development and demography of *Bactrocera dorsalis* (Diptera: Tephritidae) in China. Environ Entomol 23: 971–974.

106. Changqing Z, Kuikui W, Haidong C, Pingjun Y, Dowell RV (1994) Effect of temperature on the population growth of *Bactrocera tau* (Walker)(Dipt., Tephritidae). J Appl Entomol 117: 332–337.

107. Wilkinson JD, Daugherty DM (1970) Comparative development of *Bradysia impatiens* (Diptera: Sciaridae) under constant and variable temperatures. Ann Entomol Soc Am 63: 1079–1083.

108. Anderson G (2000) Minimum and maximum development rates of some forensically important Calliphoridae (Diptera). J Forensic Sci 45: 824.

109. Grout TG, Stoltz KC (2007) Developmental rates at constant temperatures of three economically important *Ceratitis* spp. (Diptera: Tephritidae) from Southern Africa. Environ Entomol 36: 1310–1317. doi:10.1603/0046-225X(2007)36[1310:DRACTO]2.0.CO;2.

110. Stevens MM (2003) Development and survival of *Chironomus tepperi* Skuse (Diptera: Chironomidae) at a range of constant temperatures. Aquat Insects 20: 181–188. doi:10.1076/aqin.20.3.181.4470.

111. Readshaw JL (1966) The ecology of the swede midge, *Contarinia nasturtii* (Kieff.)(Diptera, Cecidomyiidae). I.—Life-history and influence of temperature and moisture on development. Bull Entomol Res 56: 685–700.

112. Baxendale FP, Teetes G, Sharpe P, Wu H (1984) Temperature-dependent model for development of nondiapausing sorghum midges (Diptera: Cecidomyiidae). Environ Entomol 13: 1572–1576.

113. Mottram P, Kay B, Kettle D (1986) The effect of temperature on eggs and immature stages of *Culex annulirostris* Skuse (Diptera: Culicidae). Aust J Entomol 25: 131–136.

114. Reisen WK (1995) Effect of temperature on *Culex tarsalis* (Diptera: Culicidae) from the Coachella and San Joaquin valleys of California. J Med Entomol 32: 636–645.

115. Mullens BA, Rutz DA (1983) Development of immature *Culicoides variipennis* (Diptera: Ceratopogonidae) at constant laboratory temperatures. Ann Entomol Soc Am 76: 747–751.

116. Moore AD (1987) Effects of temperature and length of photophase on development and diapause in *Cystiphora schmidti* (Rübsaamen)(Diptera: Cecidomyiidae). Aust J Entomol 26: 349–354.

117. Throne JE, Eckenrode CJ (1986) Development rates for the seed maggots *Delia platura* and *D. florilega* (Diptera: Anthomyiidae). Environ Entomol 15: 1022–1027.

118. Siddiqui WH, Barlow CA (1972) Population growth of *Drosophila melanogaster* (Diptera: Drosophilidae) at constant and alternating temperatures. Ann Entomol Soc Am 65: 993–1001.

119. Gillespie DR, Opit G, Roitberg B (2000) Effects of temperature and relative humidity on development, reproduction, and predation in *Feltiella acarisuga* (Vallot)(Diptera: Cecidomyiidae). Biol Control 17: 132–138.

120. Cook IM, Spain AV (1981) Rates of development of the immature stages of the buffalo fly, *Haematobia irritans exigua* de Meijere (Diptera: Muscidae), in relation to temperature. Aust J Zool 29: 7–14.

121. Karandinos MG, Axtell RC (1967) Temperature effects on the immature stages of *Hippelates pusio*, *H. bishoppi*, and *H. pallipes* (Diptera: Chloropidae). Ann Entomol Soc Am 60: 1055–1062.

122. Leibee GL (1984) Influence of temperature on development and fecundity of *Liriomyza trifolii* (Burgess)(Diptera: Agromyzidae) on celery. Environ Entomol 13: 497–501.

123. Grassberger M, Reiter C (2001) Effect of temperature on *Lucilia sericata* (Diptera: Calliphoridae) development with special reference to the isomegalen- and isomorphen-diagram. Forensic Sci Int 120: 32–36.

124. Foster JE, Taylor PL (1975) Thermal-unit requirements for development of the Hessian fly under controlled environments. Environ Entomol 4: 195–202.

125. Moon RD (1983) Simulating developmental time of preadult face flies (Diptera: Muscidae) from air temperature records. Environ Entomol 12: 943–948.

126. Lysyk TJ, Axtell RC (1987) A simulation model of house fly (Diptera: Muscidae) development in poultry manure. Can Entomol 119: 427–437.

127. Lefebvre F, Pasquerault T (2004) Temperature-dependent development of *Ophyra aenescens* (Wiedemann, 1830) and *Ophyra capensis* (Wiedemann, 1818)(Diptera, Muscidae). Forensic Sci Int 139: 75–79.

128. Amoudi MA, Diab FM, Abou-Fannah SSM (1994) Development rate and mortality of immature *Parasarcophaga (Liopygia) ruficornis* (Diptera: Sarcophagidae) at constant laboratory temperatures. J Med Entomol 31: 168–170.

129. Byrd JH, Allen JC (2001) The development of the black blow fly, *Phormia regina* (Meigen). Forensic Sci Int 120: 79–88.

130. Russo A, Cocuzza GE, Vasta MC, Simola M, Virone G (2006) Life fertility tables of *Piophila casei* L. (Diptera: Piophilidae) reared at five different temperatures. Environ Entomol 35: 194–200. doi:10.1603/0046-225X-35.2.194.

131. Grassberger M, Reiter C (2002) Effect of temperature on development of the forensically important holarctic blow fly *Protophormia terraenovae* (Robineau-Desvoidy)(Diptera: Calliphoridae). Forensic Sci Int 128: 177–182.

132. Stevenson AB (1981) Development of the carrot rust fly, *Psila rosae* (Diptera: Psilidae), relative to temperature in the laboratory. Can Entomol 113: 569–574.

133. Barnes JK (1976) Effect of temperature on development, survival, oviposition, and diapause in laboratory populations of *Sepedon fuscipennis* (Diptera: Sciomyzidae). Environ Entomol 5: 1089–1098.

134. Lysyk TJ (1998) Relationships between temperature and life-history parameters of *Stomoxys calcitrans* (Diptera: Muscidae). J Med Entomol 35: 107–119.

135. Sweeney BW, Vannote RL (1984) Influence of food quality and temperature on life history characteristics of the parthenogenetic mayfly, *Cloeon triangulifer*. Freshw Biol 14: 621–630.

136. Sweeney BW, Vannote RL (1981) *Ephemerella* mayflies of White Clay Creek: Bioenergetic and ecological relationships among six coexisting species. Ecology 62: 1353–1369.

137. Wright LL, Mattice JS, Beauchamp JJ (1982) Effect of temperature and sex on growth patterns in nymphs of the mayfly *Hexagenia hilineata* in the laboratory. Freshw Biol 12: 535–545.

138. Simmons AM, Yeargan KV (1988) Development and survivorship of the green stink bug, *Acrosternum hilare* (Hemiptera: Pentatomidae) on soybean. Environ Entomol 17: 527–532.

139. Fargo WS, Bonjour EL (1988) Developmental rate of the squash bug, *Anasa tristis* (Heteroptera: Coreidae) at constant temperatures. Environ Entomol 17: 926–929.

140. Lu Y, Wu K, Wyckhuys KAG, Guo Y (2010) Temperature-dependent life history of the green plant bug, *Apolygus lucorum* (Meyer-Dür) (Hemiptera: Miridae). Appl Entomol Zool 45: 387–393. doi:10.1303/aez.2010.387.

141. James DG (1990) Development and survivorship of *Biprorulus bibax* (Hemiptera: Pentatomidae) under a range of constant temperatures. Environ Entomol 19: 874–877.

142. Awan MS (1988) Development and Mating Behaviour of *Oechalia Schellenbergii* (Guérin‐Méneville) and *Cermatulus Nasalis* (Westwood) (Hemiptera: Pentatomidae). Aust J Entomol 27: 183–187. doi:10.1111/j.1440-6055.1988.tb01520.x.

143. Rose DJW (1973) Laboratory observations on the biology of *Cicadulina* spp.(Horn.: Cicadellidae), with particular reference to the effects of temperature. Bull Entomol Res 62: 471–475.

144. Simonet DE, Pienkowski RL (1980) Temperature effect on development and morphometrics of the potato leafhopper. Environ Entomol 9: 798–800.

145. Harries FH, Douglass JR (1948) Bionomic studies on the beet leafhopper. Ecol Monogr: 45–79.

146. Larsen KJ, Madden LV, Nault LR (1990) Effect of temperature and host plant on the development of the blackfaced leafhopper. Entomol Exp Appl 55: 285–294.

147. Mokhtar AM, Nabhani SSA (2010) Temperature-dependent development of dubas bug, *Ommatissus lybicus* (Hemiptera: Tropiduchidae), an endemic pest of date palm, *Phoenix dactylifera*. Eur J Entomol 107.

148. Dreyer H, Baumgärtner J (1996) Temperature influence on cohort parameters and demographic characteristics of the two cowpea coreids *Clavigralla tomentosicollis* and *C. shadabi*. Entomol Exp Appl 78: 201–213.

149. Neal JW, Douglass LW (1990) Seasonal dynamics and the effect of temperature in *Corythucha cydoniae* (Heteroptera: Tingidae). Environ Entomol 19: 1299–1304.

150. Foley DH, Pyke BA (1985) Developmental time of *Creontiades dilutus* (Ståal)(Hemiptera: Miridae) in relation to temperature. Aust J Entomol 24: 125–127.

151. Kim D-S, Riedl H (2005) Effect of temperature on development and fecundity of the predaceous plant bug *Deraeocoris brevis* reared on *Ephestia kuehniella* eggs. Biocontrol 50: 881–897. doi:10.1007/s10526-005-5380-x.

152. Gillespie DR, Sanchez JAS, McGregor RR (2004) Cumulative temperature requirements and development thresholds in two populations of *Dicyphus hesperus* (Hemiptera: Miridae). Can Entomol 136: 675–683.

153. Toscano NC, Stern VM (1976) Development and reproduction of *Euschistus conspersus* at different temperatures. Ann Entomol Soc Am 69: 839–840.

154. Dunbar DM, Bacon OG (1972) Influence of temperature on development and reproduction of *Geocoris atricolor*, *G. pallens*, and *G. punctipes* (Heteroptera: Lygaeidae) from California. Environ Entomol 1: 596–599.

155. Spence JR, Spence DH, Scudder GGE (1980) The effects of temperature on growth and development of water strider species (Heteroptera: Gerridae) of central British Columbia and implications for species packing. Can J Zool 58: 1813–1820.

156. Nielsen AL, Hamilton GC, Matadha D (2008) Developmental rate estimation and life table analysis for *Halyomorpha halys* (Hemiptera: Pentatomidae). Environ Entomol 37: 348–355. doi:10.1603/0046-225X(2008)37[348:DREALT]2.0.CO;2.

157. Diaz R, Overholt WA, Cuda JP, Pratt PD, Fox A (2008) Temperature-dependent development, survival, and potential distribution of *Ischnodemus variegatus* (Hemiptera: Blissidae), a herbivore of West Indian marsh grass. Ann Entomol Soc Am 101: 604–612. doi:10.1603/0013-8746(2008)101[604:TDSAPD]2.0.CO;2.

158. Morimoto S, Imamura T, Visarathanonth P, Miyanoshita A (2007) Effects of temperature on the development and reproduction of the predatory bug *Joppeicus paradoxus* Puton (Hemiptera: Joppeicidae) reared on *Tribolium confusum* eggs. Biol Control 40: 136–141.

159. Parajulee MN, Phillips TW, Throne JE, Nordheim EV (1995) Life history of immature *Lyctocoris campestris* (Hemiptera: Anthocoridae): effects of constant temperatures and relative humidities. Environ Entomol 24: 889–897.

160. Bommireddy PL, Parajulee MN, Porter DO (2004) Influence of constant temperatures on life history of immature *Lygus elisus* (Hemiptera: Miridae). Environ Entomol 33: 1549–1553. doi:10.1603/0046-225X-33.6.1549.

161. Champlain RA, Butler Jr. GD (1967) Temperature effects on development of the egg and nymphal stages of *Lygus hesperus* (Hemiptera: Miridae). Ann Entomol Soc Am 60: 519–521.

162. Khattat AR, Stewart RK (1977) Development and survival of *Lygus lineolaris* exposed to different laboratory rearing conditions. Ann Entomol Soc Am 70: 274–278.

163. Perdikis DC, Lykouressis DP (2002) Thermal requirements for development of the polyphagous predator *Macrolophus pygmaeus* (Hemiptera: Miridae). Environ Entomol 31: 661–667. doi:10.1603/0046-225X-31.4.661.

164. Braman SK, Yeargan KV (1988) Comparison of developmental and reproductive rates of *Nabis americoferus*, *N. roseipennis*, and *N. rufusculus* (Hemiptera: Nabidae). Ann Entomol Soc Am 81: 923–930.

165. Samson PR, Blood PRB (1979) Biology and temperature relationships of *Chrysopa* sp., *Micromus tasmaniae* and *Nabis capsiformis*. Entomol Exp Appl 25: 253–259.

166. Kehat M, Wyndham M (1972) The influence of temperature on development, longevity, and fecundity in the Rutherglen bug, *Nysius vinitor* (Hemiptera: Lygaeidae). Aust J Zool 20: 67–78.

167. Naresh JS, Smith CM (1983) Development and survival of rice stink bugs (Hemiptera: Pentatomidae) reared on different host plants at four temperatures. Environ Entomol 12: 1496–1499.

168. Cocuzza G, De Clercq P, Van De Veire M, De Cock A, Degheele D, et al. (1997) Reproduction of *Orius laevigatus* and *Orius albidipennis* on pollen and *Ephestia kuehniella* eggs. Entomol Exp Appl 82: 101–104.

169. Isenhour DJ, Yeargan KV (1981) Effect of temperature on the development of *Orius insidiosus*, with notes on laboratory rearing. Ann Entomol Soc Am 74: 114–116.

170. Nagai K, Yano E (1999) Effects of temperature on the development and reproduction of *Orius sauteri* (Poppius)(Heteroptera: Anthocoridae), a predator of *Thrips palmi* Karny (Thysanoptera: Thripidae). Appl Entomol Zool 34: 223–229.

171. Ohta I (2001) Effect of temperature on development of *Orius strigicollis* (Heteroptera: Anthocoridae) fed on *Frankliniella occidentalis* (Thysanoptera: Thripidae). Appl Entomol Zool 36: 483–488.

172. Butler Jr. GD (1966) Development of several predaceous Hemiptera in relation to temperature. J Econ Entomol 59: 1306–1307.

173. Stoner A, Metcalfe AM, Weeks RE (1974) Development of *Podisus acutissimus* in relation to constant temperature. Ann Entomol Soc Am 67: 718–719.

174. Drummond FA, James RL, Casagrande RA, Faubert HH (1984) Development and survival of *Podisus maculiventris* (Say)(Hemiptera: Pentatomidae), a predator of the Colorado potato beetle (Coleoptera: Chrysomelidae). Environ Entomol 13: 1283–1286.

175. Medeiros RS, Ramalho FS, Serrão JE, Zanuncio JC (2004) Estimative of *Podisus nigrispinus* (Dallas)(Heteroptera: Pentatomidae) development time with non linear models. Neotrop Entomol 33: 141–148.

176. De Clercq P, Degheele D (1992) Development and survival of *Podisus maculiventris* (Say) and *Podisus sagitta* (Fab.)(Heteroptera: Pentatomidae) at various constant temperatures. Can Entomol 124: 125–133.

177. James DG (1992) Effect of temperature on development and survival of *Pristhesancus plagipennis* [Hem.: Reduviidae]. BioControl 37: 259–264.

178. Gaylor MJ, Sterling WL (1975) Effects of temperature on the development, egg production, and survival of the cotton fleahopper, *Pseudatomoscelis seriatus*. Environ Entomol 4: 487–490.

179. Lopatina EB, Balashov SV, Kipyatkov VE (2007) First demonstration of the influence of photoperiod on the thermal requirements for development in insects and in particular the linden-bug, *Pyrrhocoris apterus* (Heteroptera: Pyrrhocoridae). Eur J Entomol 104: 23–31.

180. Butler Jr. GD (1970) Temperature and the development of *Spanagonicus albofasciatus* and *Rhinacloa forticornis*. J Econ Entomol 63: 669–670.

181. Kim H, Baek S, Kim S, Lee S-Y, Lee J-H (2009) Temperature-dependent development and oviposition models of *Riptortus clavatus* (Thunberg) (Hemiptera: Alydidae). Appl Entomol Zool 44: 515–523. doi:10.1303/aez.2009.515.

182. Neal JW, Douglass LW (1988) Development, oviposition rate, longevity, and voltinism of *Stephanitis pyrioides* (Heteroptera: Tingidae), an adventive pest of *Azalea*, at three temperatures. Environ Entomol 17: 827–831.

183. Arbogast RT (1975) Population growth of *Xylocoris flavipes*: influence of temperature and humidity. Environ Entomol 4: 825–831.

184. Summers CG, Coviello RL, Gutierrez AP (1984) Influence of constant temperatures on the development and reproduction of *Acyrthosiphon kondoi* (Homoptera: Aphididae). Environ Entomol 13: 236–242.

185. Morgan D, Walters KFA, Aegerter JN (2001) Effect of temperature and cultivar on pea aphid, *Acyrthosiphon pisum* (Hemiptera: Aphididae) life history. Bull Entomol Res 91: 47–52.

186. Dowell RV, Fitzpatrick GE (1978) Effects of temperature on the growth and survivorship of the citrus blackfly (Homoptera: Aleyrodidae). Can Entomol 110: 1347–1350.

187. Iheagwam EU (1978) Effects of temperature on development of the immature stages of the cabbage whitefly, *Aleyrodes proletella* (Homoptera: Aleyrodidae). Entomol Exp Appl 23: 91–95.

188. Kennett CE, Hoffmann RW (1985) Seasonal development of the California red scale (Homoptera: Diaspididae) in San Joaquin Valley citrus based on degree-day accumulation. J Econ Entomol 78: 73–79.

189. Xia JY, Werf W, Rabbinge R (1999) Influence of temperature on bionomics of cotton aphid, *Aphis gossypii*, on cotton. Entomol Exp Appl 90: 25–35.

190. Wang K, Tsai JH, Harrison NA (1997) Influence of temperature on development, survivorship, and reproduction of buckthorn aphid (Homoptera: Aphididae). Ann Entomol Soc Am 90: 62–68.

191. Carroll DP, Hoyt SC (1986) Some effects of parental rearing conditions and age on progeny birth weight, growth, development, and reproduction in the apple aphid, *Aphis pomi* (Homoptera: Aphididae). Environ Entomol 15: 614–619.

192. Wang K, Tsai JH (1996) Temperature effect on development and reproduction of silverleaf whitefly (Homoptera: Aleyrodidae). Ann Entomol Soc Am 89: 375–384.

193. Powell DA, Bellows TS (1992) Preimaginal development and survival of *Bemisia tabaci* on cotton and cucumber. Environ Entomol 21: 359–363.

194. Kairo MTK, Murphy ST (1999) Temperature and plant nutrient effects on the development, survival and reproduction of *Cinara* sp. nov., an invasive pest of cypress trees in Africa. Entomol Exp Appl 92: 147–156.

195. Liu YH, Tsai JH (2000) Effects of temperature on biology and life table parameters of the Asian citrus psyllid, *Diaphorina citri* Kuwayama,(Homoptera: Psyllidae). Ann Appl Biol 137: 201–206.

196. Wellings PW (1981) The effect of temperature on the growth and reproduction of two closely related aphid species on sycamore. Ecol Entomol 6: 209–214.

197. Blommers LHM, Helsen HHM, Vaal FWNM (2004) Life history data of the rosy apple aphid *Dysaphis plantaginea* (Pass.) (Homopt., Aphididae) on plantain and as migrant to apple. J Pest Sci 77. Available: http://springerlink.metapress.com/openurl.asp?genre=article&id=doi:10.1007/s10340-004-0046-5. Accessed 26 September 2011.

198. Asante S, Danthanarayana W, Heatwole H (1991) Bionomics and population growth statistics of apterous virginoparae of woolly apple aphid, *Eriosoma lanigerum*, at constant temperatures. Entomol Exp Appl 60: 261–270.

199. Patil NG, Baker PS, Pollard GV (1994) Life history parameters of the Leucaena psyllid *Heteropsylla cubana* (Crawford)(Homoptera: Psyllidae) under various temperature and relative humidity regimes. Insect Sci Its Appl 15: 293–300.

200. Shu-Sheng L, Hughes RD (1987) The influence of temperature and photoperiod on the development, survival and reproduction of the sowthistle aphid, *Hyperomyzus lactucae*. Entomol Exp Appl 43: 31–38.

201. Liu TX, Yue B (2001) Comparison of some life history parameters between alate and apterous forms of turnip aphid (Homoptera: Aphididae) on cabbage under constant temperatures. Fla Entomol: 239–242.

202. Kieckhefer RW, Elliott NC, Walgenbach DD (1989) Effects of constant and fluctuating temperatures on developmental rates and demographic statistics of the English grain aphid (Homoptera: Aphididae). Ann Entomol Soc Am 82: 701–706.

203. Barlow CA (1962) The influence of temperature on the growth of experimental populations of *Myzus persicae* (Sulzer) and *Macrosiphum euphorbiae* (Thomas)(Aphididae). Can J Zool 40: 145–156.

204. Dean GJ (1974) Effect of temperature on the cereal aphids *Metopolophium dirhodum* (Wlk.), *Rhopalosiphum padi* (L.) and *Macrosiphum avenue* (F.)(Hem., Aphididae). Bull Entomol Res 63: 401–409.

205. Lema KM, Herren HR (1985) The influence of constant temperature on population growth rates of the cassava mealybug, *Phenacoccus manihoti*. Entomol Exp Appl 38: 165–169.

206. Hwang J, Hsieh F, Hung C, Chu Y (1988) Life history and the effect of temperature on population growth parameters of *Planococcus pacificus* on Guava. Plant Prot Bull 30: 157–174.

207. McMullen RD, Jong C (1977) Effect of temperature on developmental rate and fecundity of the pear psylla, *Psylla pyricola* (Homoptera: Psyllidae). Can Entomol 109: 165–169.

208. Tsai JH, Liu YH (1998) Effect of temperature on development, survivorship, and reproduction of rice root aphid (Homoptera: Aphididae). Environ Entomol 27: 662–666.

209. Rae DJ, De’ath G (1991) Influence of constant temperature on development, survival and fecundity of sugarcane mealybug, *Saccharicoccus sacchari* (Cockerell)(Hemiptera, Pseudococcidae). Aust J Zool 39: 105–122.

210. Acreman S, Dixon A (1989) The effects of temperature and host quality on the rate of increase of the grain aphid (*Sitobion avenae*) on wheat. Ann Appl Biol 115: 3–9.

211. Butler Jr. GD (1967) Development of the banded-wing whitefly at different temperatures. J Econ Entomol 60: 877–878.

212. Greenberg SM, Legaspi BC, Jones WA, Enkegaard A (2000) Temperature-dependent life history of *Eretmocerus eremicus* (Hymenoptera: Aphelinidae) on two whitefly hosts (Homoptera: Aleyrodidae). Environ Entomol 29: 851–860. doi:10.1603/0046-225X-29.4.851.

213. Herrera CJ, Driesche RG, Bellotti AC (1989) Temperature-dependent growth rates for the cassava mealybug, *Phenacoccus herreni*, and two of its encyrtid parasitoids, *Epidinocarsis diversicornis* and *Acerophagus coccois* in Colombia. Entomol Exp Appl 50: 21–27.

214. De Vis RMJ, Fuentes LE, Van Lenteren JC (2002) Life history of *Amitus fuscipennis* (Hym., Platygastridae) as parasitoid of the greenhouse whitefly *Trialeurodes vaporariorum*(Hom., Aleyrodidae) on tomato as function of temperature. J Appl Entomol 126: 24–33.

215. Pandey RR, Johnson MW (2006) Physiological and morphological development of *Anagyrus ananatis* at constant temperatures. BioControl 51: 585–601.

216. Daane KM, Malakar-Kuenen RD, Walton VM (2004) Temperature-dependent development of *Anagyrus pseudococci* (Hymenoptera: Encyrtidae) as a parasitoid of the vine mealybug, *Planococcus ficus* (Homoptera: Pseudococcidae). Biol Control 31: 123–132.

217. Cardona C, Oatman E (1975) Biology and Physical Ecology of Apanteles Subandinus Blanchard (Hymenoptera: Braconidae), with Notes on Temperature Responeses on Apanteles Scutellaris Muesebeck and Its Hosts, the Potato Tuberworm. University of California, Division of Agriculture and Natural Resources.

218. Lee J-H, Elliott NC (1998) Comparison of developmental responses to temperature in *Aphelinus asychis* (Walker) from two different geographic regions. Southwest Entomol 23: 77–82.

219. Tang YQ, Yokomi RK (1995) Temperature-dependent development of three hymenopterous parasitoids of aphids (Homoptera: Aphididae) attacking citrus. Environ Entomol 24: 1736–1740.

220. Force DC, Messenger PS (1964) Duration of development, generation time, and longevity of three Hymenopterous parasites of *Therioaphis maculata*, reared at various constant temperatures. Ann Entomol Soc Am 57: 405–413.

221. Zamani AA, Talebi A, Fathipour Y, Baniameri V (2007) Effect of temperature on life history of *Aphidius colemani* and *Aphidius matricariae* (Hymenoptera: Braconidae), two parasitoids of *Aphis gossypii* and *Myzus persicae* (Homoptera: Aphididae). Environ Entomol 36: 263–271. doi:10.1603/0046-225X-36.2.263.

222. Abdelrahman I (1974) Growth, development and innate capacity for increase in *Aphytis chrysomphali* Mercet and *A. melinus* DeBach, parasites of California red scale, *Aonidiella aurantii* (Mask.), in relation to temperature. Aust J Zool 22: 213–230.

223. Ramalho FS, Wanderley PA, Malaquias JB, Rodrigues KCV, Souza JVS, et al. (2009) Temperature-dependent development rates of *Bracon vulgaris*, a parasitoid of boll weevil. Phytoparasitica 37: 17–25.

224. Morales-Ramos JA, Cate JR (1993) Temperature-dependent developmental rates of *Catolaccus grandis* (Hymenoptera: Pteromalidae). Environ Entomol 22: 226–233.

225. Horne P, Horne J (1991) The effects of temperature and host density on the development and survival of *Copidosoma koehleri*. Entomol Exp Appl 59: 289–292.

226. Mbapila JC, Overholt WA (2001) Comparative development, longevity and population growth of exotic and native parasitoids of Lepidopteran cereal stemborers in Kenya. Bull Entomol Res 91: 347–354.

227. Jones JM, Stephen FM (1994) Effect of temperature on development of hymenopterous parasitoids of *Dendroctonus frontalis* (Coleoptera: Scolytidae). Environ Entomol 23: 457–463.

228. Golizadeh A, Kamali K, Fathipour Y, Abbasipour H (2008) Life table and temperature-dependent development of *Diadegma anurum* (Hymenoptera: Ichneumonidae) on its host *Plutella xylostella* (Lepidoptera: Plutellidae). Environ Entomol 37: 38–44. doi:10.1603/0046-225X(2008)37[38:LTATDO]2.0.CO;2.

229. Mehrnejad MR (2003) The influence of host species on some biological and behavioural aspects of *Dibrachys boarmiae* (Hymenoptera: Pteromalidae), parasitoid of *Kermania pistaciella* (Lepidoptera: Tineidae). Biocontrol Sci Technol 13: 219–229. doi:10.1080/0958315021000073484.

230. Haghani M, Fathipour Y, Talebi AA, Baniameri V (2006) Temperature-dependent development of *Diglyphus isaea* (Hymenoptera: Eulophidae) on *Liriomyza sativae* (Diptera: Agromyzidae) on cucumber. J Pest Sci 80: 71–77. doi:10.1007/s10340-006-0154-5.

231. Obrycki JJ, Tauber MJ, Tauber CA, Gollands B (1987) Developmental responses of the Mexican biotype of *Edovum puttleri* (Hymenoptera: Eulophidae) to temperature and photoperiod. Environ Entomol 16: 1319–1323.

232. Matadha D, Hamilton GC, Lashomb JH (2004) Effect of temperature on development, fecundity, and life table parameters of *Encarsia citrina* Craw (Hymenoptera: Aphelinidae), a parasitoid of *Euonymus* scale, *Unaspis euonymi* (Comstock), and *Quadraspidiotus perniciosus* (Comstock) (Homoptera: Diaspididae). Environ Entomol 33: 1185–1191. doi:10.1603/0046-225X-33.5.1185.

233. Shishehbor P, Brennan PA (1996) Functional response of *Encarsia formosa* (Gahan) parasitizing castor whitefly, *Trialeurodes ricini* Misra (Hom., Aleyrodidae). J Appl Entomol 120: 297–299.

234. Avilla J, Copland MJW (1988) Development rate, number of mature oocytes at emergence and adult size of *Encarsia tricolor* at constant and variable temperatures. BioControl 33: 289–298.

235. Cohen MB, Mackauer M (1987) Intrinsic rate of increase and temperature coefficients of the aphid parasite *Ephedrus californicus* Baker (Hymenoptera: Aphididae). Can Entomol 119: 231–237.

236. Tullett AG, Hart AJ, Worland MR, Bale JS (2004) Assessing the effects of low temperature on the establishment potential in Britain of the non-native biological control agent *Eretmocerus eremicus*. Physiol Entomol 29: 363–371.

237. Butler Jr. GD, Schmidt KM (1985) *Goniozus legneri* (Hymenoptera: Bethylidae): development, oviposition, and longevity in relation to temperature. Ann Entomol Soc Am 78: 373–375.

238. Abe Y, Tahara M (2003) Daily progeny production and thermal influence on development and adult longevity of the leafminer parasitoid, *Gronotoma micromorpha* (Hym., Eucoilidae). J Appl Entomol 127: 477–480.

239. Tingle C, Copland MJW (1988) Predicting development of the mealybug parasitoids *Anagyrus pseudococci*, *Leptomastix dactylopii* and *Leptomastidae abnormis* under glasshouse conditions. Entomol Exp Appl 46: 19–28.

240. Dittrick LE, Chiang H (1982) Developmental characteristics of *Macrocentrus grandii* as influenced by temperature and instar of its host, the European corn borer. J Insect Physiol 28: 47–52.

241. Krugner R, Daane KM, Lawson AB, Yokota GY (2007) Temperature-dependent development of *Macrocentrus iridescens* (Hymenoptera: Braconidae) as a parasitoid of the obliquebanded leafroller (Lepidoptera: Tortricidae): Implications for field synchrony of parasitoid and host. Biol Control 42: 110–118.

242. Lysyk TJ (2000) Relationships between temperature and life history parameters of *Muscidifurax raptor* (Hymenoptera: Pteromalidae). Environ Entomol 29: 596–605.

243. Lysyk TJ (2001) Relationships between temperature and life history parameters of *Muscidifurax raptorellus* (Hymenoptera: Pteromalidae). Environ Entomol 30: 982–992. doi:10.1603/0046-225X-30.5.982.

244. Lysyk TJ (2001) Relationships between temperature and life history parameters of *Muscidifurax zaraptor* (Hymenoptera: Pteromalidae). Environ Entomol 30: 147–156. doi:10.1603/0046-225X-30.1.147.

245. Grassberger M, Frank C (2003) Temperature-related development of the parasitoid wasp *Nasonia vitripenni*s as forensic indicator. Med Vet Entomol 17: 257–262.

246. Wang X, Liu S, Guo S, Lin W (1999) Effects of host stages and temperature on population parameters of *Oomyzus sokolowskii*, a larval-pupal parasitoid of *Plutella xylostella*. BioControl 44: 391–403.

247. Roeser-Mueller K, Strohm E, Kaltenpoth M (2010) Larval rearing temperature influences amount and composition of the marking pheromone of the male beewolf, *Philanthus triangulum*. J Insect Sci 10: 74.

248. Campbell A, Mackauer M (1975) Thermal constants for development of the pea aphid (Homoptera: Aphididae) and some of its parasites. Can Entomol 107: 419–423.

249. Infante F (2000) Development and population growth rates of *Prorops nasuta* (Hym., Bethylidae) at constant temperatures. J Appl Entomol 124: 343–348.

250. Mann JA, Axtell RC, Stinner RE (1990) Temperature-dependent development and parasitism rates of four species of Pteromalidae (Hymenoptera) parasitoids of house fly (*Musca domestica*) pupae. Med Vet Entomol 4: 245–253.

251. Geden CJ (1997) Development models for the filth fly parasitoids *Spalangia gemina*, *S. cameroni*, and *Muscidifurax raptor* (Hymenoptera: Pteromalidae) under constant and variable temperatures. Biol Control 9: 185–192.

252. Ruberson JR, Tauber CA, Tauber MJ (1995) Developmental effects of host and temperature on *Telenomus* spp (Hymenoptera: Scelionidae) parasitizing chrysopid eggs. Biol Control 5: 245–250.

253. Cave RD, Gaylor MJ (1988) Influence of temperature and humidity on development and survival of *Telenomus reynoldsi* (Hymenoptera: Scelionidae) parasitizing *Geocoris punctipes* (Heteroptera: Lygaeidae) eggs. Ann Entomol Soc Am 81: 278–285.

254. Jubb GL, Watson TF (1971) Development of the egg parasite *Telenomus utahensis* in two pentatomid hosts in relation to temperature and host age. Ann Entomol Soc Am 64: 202–205.

255. Haile AT, Hassan SA, Ogol CKPO, Baumgärtner J, Sithanantham S, et al. (2002) Temperature-dependent development of four egg parasitoid *Trichogramma* species (Hymenoptera: Trichogrammatidae). Biocontrol Sci Technol 12: 555–567. doi:10.1080/0958315021000016225.

256. Lawrence RK, Houseweart MW, Jennings DT, Southard SG, Halteman WA (1985) Development rates of *Trichogramma minutum* (Hymenoptera: Trichogrammatidae) and implications for timing augmentative releases for suppression of egg populations of *Choristoneura fumiferana* (Lepidoptera: Tortricidae). Can Entomol 117: 557–563.

257. Wang B, Ferro DN, Wu J, Wang S (2004) Temperature-dependent development and oviposition behavior of *Trichogramma ostriniae* (Hymenoptera: Trichogrammatidae), a potential biological control agent for the european corn borer (Lepidoptera: Crambidae). Environ Entomol 33: 787–793. doi:10.1603/0046-225X-33.4.787.

258. Pratissoli D, Fernandes OA, Zanuncio JC, Pastori PL (2004) Fertility life table of *Trichogramma pretiosum* and *Trichogramma acacioi* (Hymenoptera: Trichogrammatidae) on *Sitotroga cerealella* (Lepidoptera: Gelechiidae) eggs at different constant temperatures. Ann Entomol Soc Am 97: 729–731. doi:10.1603/0013-8746(2004)097[0729:FLTOTP]2.0.CO;2.

259. Lysyk TJ (1998) Relationships between temperature and life history parameters of *Trichomalopsis sarcophagae* (Hymenoptera: Pteromalidae). Environ Entomol 27: 488–498.

260. Torres JB, Musolin DL, Zanuncio JC (2002) Thermal requirements and parasitism capacity of *Trissolcus brochymenae* (Ashmead) (Hymenoptera: Scelionidae) under constant and fluctuating temperatures, and assessment of development in field conditions. Biocontrol Sci Technol 12: 583–593. doi:10.1080/0958315021000016243.

261. James DG, Warren GN (1991) Effect of temperature on development, survival, longevity and fecundity of *Trissolcus oenone* Dodd (Hymenoptera: Scelionidae). Aust J Entomol 30: 303–306.

262. Huis A, Arendse PW, Schilthuizen M, Wiegers PP, Heering H, et al. (1994) *Uscana lariophaga*, egg parasitoid of bruchid beetle storage pests of cowpea in West Africa: the effect of temperature and humidity. Entomol Exp Appl 70: 41–53.

263. Damos PT, Savopoulou-Soultani M (2008) Temperature-dependent bionomics and modeling of *Anarsia lineatella* (Lepidoptera: Gelechiidae) in the laboratory. J Econ Entomol 101: 1557–1567. doi:10.1603/0022-0493(2008)101[1557:TBAMOA]2.0.CO;2.

264. McClay AS, Hughes RB (1995) Effects of temperature on developmental rate, distribution, and establishment of *Calophasia lunula* (Lepidoptera: Noctuidae), a biocontrol agent for toadflax (*Linaria* spp). Biol Control 5: 368–377.

265. Jones R, Rienks J, Wilson L, Lokkers C, Churchill T (1987) Temperature, development and survival in monophagous and polyphagous tropical pierid butterflies. Aust J Zool 35: 235–246.

266. Aghdam HR, Fathipour Y, Radjabi G, Rezapanah M (2009) Temperature-dependent development and temperature thresholds of codling moth (Lepidoptera: Tortricidae) in Iran. Environ Entomol 38: 885–895. doi:10.1603/022.038.0343.

267. Rodriguez-del-Bosque LA, Smith Jr JW, Browning HW (1989) Development and life-fertility tables for *Diatraea lineolata* (Lepidoptera: Pyralidae) at constant temperatures. Ann Entomol Soc Am 82.

268. Sandhu HS, Nuessly GS, Webb SE, Cherry RH, Gilbert RA (2010) Temperature-dependent development of *Elasmopalpus lignosellus* (Lepidoptera: Pyralidae) on sugarcane under laboratory conditions. Environ Entomol 39: 1012–1020. doi:10.1603/EN09284.

269. Siddiqui WH, Barlow CA (1973) Population growth of *Anagasta kuehniella* (Lepidoptera: Pyralidae) at constant and alternating temperatures. Ann Entomol Soc Am 66: 579–585.

270. Manrique V, Cuda JP, Overholt WA, Diaz R (2008) Temperature-dependent development and potential distribution of *Episimus utilis* (Lepidoptera: tortricidae), a candidate biological control agent of brazilian peppertree (Sapindales: Anacardiaceae) in Florida. Environ Entomol 37: 862–870. doi:10.1603/0046-225X(2008)37[862:TDAPDO]2.0.CO;2.

271. Ashamo M, Odeyemi O (2001) Effect of rearing temperature on the fecundity and development of *Euzopherodes vapidella* Mann (Lepidoptera: Pyralidae), a pest of stored yam. J Stored Prod Res 37: 253–261.

272. Sharpe PJH, Schoolfield RM, Butler Jr GD (1981) Distribution model of *Heliothis zea* (Lepidoptera: Noctuidae) development times. Can Entomol 113: 845–856.

273. Gomi T, Inudo M, Yamada D (2003) Local divergence in developmental traits within a trivoltine area of *Hyphantria cunea* Drury (Lepidoptera: Arctiidae). Entomol Sci 6: 71–75.

274. Bailey CG (1976) Temperature effects on non-diapause development in *Mamestra configurata* (Lepidoptera: Noctuidae). Can Entomol 108: 1339–1344.

275. Adati T, Nakamura S, Tamò M, Kawazu K (2004) Effect of temperature on development and survival of the legume pod borer, *Maruca vitrata* (Fabricius)(Lepidoptera: Pyralidae) reared on a semi-synthetic diet. Appl Entomol Zool 39: 139–145.

276. Smith AM (1984) Larval instar determination and temperature-development studies of immature stages of the common armyworm, *Mythimna convecta* (walker)(Lepidoptera: Noctuidae). Aust J Entomol 23: 91–97.

277. Raina AK, Bell RA, Carlson RB (1977) Influence of temperature on development of an India strain of the pink bollworm in the laboratory and observations on fecundity. Ann Entomol Soc Am 70: 628–630.

278. Wakisaka S, Tsukuda R, Nakasuji F (1992) Effects of natural enemies, rainfall, temperature and host plants on survival and reproduction of the diamondback moth and other crucifer pests. Diamondback Moth Crucif Pests Proc Second Int Work Ed NS Talekar: 16–36.

279. Hansen LS, Skovgård H, Hell K (2004) Life table study of *Sitotroga cerealella* (Lepidoptera: Gelichiidae), a strain from West Africa. J Econ Entomol 97: 1484–1490. doi:10.1603/0022-0493-97.4.1484.

280. Tauber CA, Johnson JB, Tauber MJ (1992) Larval and developmental characteristics of the endemic Hawaiian lacewing, *Anomalochrysa frater* (Neuroptera: Chrysopidae). Ann Entomol Soc Am 85: 200–206.

281. Tauber MJ, Tauber CA, Hilton TW (2006) Life history and reproductive behavior of the endemic Hawaiian *Anomalochrysa hepatica* (Neuroptera: Chrysopidae): A comparative approach. Eur J Entomol 103: 327–336.

282. Butler Jr. GD, Ritchie Jr. PL (1970) Development of *Chrysopa carnea* at constant and fluctuating temperatures. J Econ Entomol 63: 1028–1030.

283. Tauber MJ, Tauber CA (1974) Thermal accumulations, diapause, and oviposition in a conifer-inhabiting predator, *Chrysopa harrisii* (Neuroptera). Can Entomol 106: 969–978.

284. Tauber CA, Tauber MJ, Nechols JR (1987) Thermal requirements for development in *Chrysopa oculata*: a geographically stable trait. Ecology: 1479–1487.

285. Leggott M, Pritchard G (1985) The effect of temperature on rate of egg and larval development in populations of *Argia vivida* Hagen (Odonata: Coenagrionidae) from habitats with different thermal regimes. Can J Zool 63: 2578–2582.

286. Waringer JA, Humpesch UH (1984) Embryonic development, larval growth and life cycle of *Coenagrion puella* (Odonata: Zygoptera) from an Austrian pond. Freshw Biol 14: 385–399.

287. Busvine JR (1955) Simple methods for rearing the cricket (*Gryllulus domesticus* L.) with some observations on speed of development at different temperatures. Proceedings of the Royal Entomological Society of London. Series A, General Entomology. Vol. 30. pp. 15–18.

288. Walters RJ, Hassall M (2006) The temperature-size rule in ectotherms: may a general explanation exist after all? Am Nat 167: 510–523.

289. Gregg P (1983) Development of the Australian plague locust, *Chortoicetes terminifera*, in relation to weather I. Effects of constant temperature and humidity. Aust J Entomol 22: 247–251.

290. Sturm R (2002) Einfluss der temperatur auf die embryonal-und larvalentwicklung bei verschiedenen grillenarten (Insecta: Orthoptera). Linz Biol Beitr 34: 485–502.

291. Bellinger RG, Pienkowski RL (1989) Polymorphic development in relation to the life history of *Melanoplus femurrubrum* (Orthoptera: Acrididae). Ann Entomol Soc Am 82: 166–171.

292. Parker JR (1930) Some effects of temperature and moisture upon *Melanoplus mexicanus mexicanus* Saussure and *Camnula pellucida* Scudder (Orthoptera). Bull Mont Agric Exp Stn.

293. Fielding DJ (2004) Developmental time of *Melanoplus sanguinipes* (Orthoptera: Acrididae) at high latitudes. Environ Entomol 33: 1513–1522. doi:10.1603/0046-225X-33.6.1513.

294. Whitman DW (1986) Developmental thermal requirements for the grasshopper *Taeniopoda eques* (Orthoptera: Aerididae). Ann Entomol Soc Am 79: 711–714.

295. Park YS, Kwon TS, Kim CS, Park JD, Kim JK (2003) Effect of temperatures on the development of the stick insect, *Baculum elongatus* (Phasmida: Phasmidae) and the life cycle. J Korean For Soc.

296. Wang J-J, Tsai JH, Zhao Z-M, Li L-S (2000) Development and reproduction of the psocid *Liposcelis bostrychophila* (Psocoptera: Liposcelididae) as a function of temperature. Ann Entomol Soc Am 93: 261–270. doi:10.1603/0013-8746(2000)093[0261:DAROTP]2.0.CO;2.

297. Wang J-J, Ren Y, Wei X-Q, Dou W (2009) Development, survival, and reproduction of the psocid *Liposcelis paeta* (Psocoptera: Liposcelididae) as a function of temperature. J Econ Entomol 102: 1705–1713. doi:10.1603/029.102.0439.

298. Gautam SG, Opit GP, Giles KL (2010) Population growth and development of the Psocid *Liposcelis rufa* (Psocoptera: Liposcelididae) at constant temperatures and relative humidities. J Econ Entomol 103: 1920–1928. doi:10.1603/EC10127.

299. Silverman J, Rust MK, Reierson DA (1981) Influence of temperature and humidity on survival and development of the cat flea, *Ctenocephalides felis* (Siphonaptera: Pulicidae). J Med Entomol 18: 78–83.

300. Amin OM, Jun L, Shangjun L, Yumei Z, Lianzhi S (1993) Development and longevity of *Nosopsyllus laeviceps kuzenkovi* (Siphonaptera) from Inner Mongolia under laboratory conditions. J Parasitol: 193–197.

301. Cooke BD, Skewes MA (1988) The effects of temperature and humidity on the survival and development of the European rabbit flea, *Spilopsyllus cuniculi* (Dale). Aust J Zool 36: 649–659.

302. Sharif M (1949) Effects of constant temperature and humidity on the development of the larvae and the pupae of the three Indian species of *Xenopsylla* (Insecta: Siphonaptera). Philos Trans R Soc Lond B 233: 581–633.

303. Lowry VK, Smith JW, Mitchell FL (1992) Life-fertility tables for *Frankliniella fusca* (Hinds) and *F. occidentalis* (Pergande)(Thysanoptera: Thripidae) on peanut. Ann Entomol Soc Am 85: 744–754.

304. Nondillo A, Redaelli LR, Botton M, Pinent SMJ, Gitz R (2008) Exigências térmicas e estimativa do número de gerações anuais de *Frankliniella occidentalis* (Pergande)(Thysanoptera: Thripidae) em morangueiro. Neotrop Entomol 37: 646–650.

305. Paine TD (1992) Cuban laurel thrips (Thysanoptera: Phlaeothripidae) biology in southern California: seasonal abundance, temperature dependent development, leaf suitability, and predation. Ann Entomol Soc Am 85: 164–172.

306. Gitonga LM, Lohr B, Overholt WA, Magambo JK, Mueke JM (2002) Temperature-dependent development of *Megalurothrips sjostedti* and *Frankliniella occidentalis* (Thysanoptera: Thripidae). Afr Entomol 10: 325–331.

307. Hoddle MS (2002) Developmental and reproductive biology of *Scirtothrips perseae* (Thysanoptera: Thripidae): a new avocado pest in California. Bull Entomol Res 92: 279–286.

308. Coville PL, Allen WW (1977) Life table and feeding habits of *Scolothrips sexmaculatus* (Thysanoptera: Thripidae). Ann Entomol Soc Am 70: 11–16.

309. Stacey DA, Fellowes MDE (2002) Temperature and the developmental rates of thrips: Evidence for a constraint on local adaptation? Eur J Entomol 99: 399–404.

310. Teulon DAJ, Penman DR (1991) Effects of temperature and diet on oviposition rate and development time of the New Zealand flower thrips, *Thrips obscuratus*. Entomol Exp Appl 60: 143–155.

311. Tsai JH, Yue B, Webb SE, Funderburk JE, Hsu HT (1995) Effects of host plant and temperature on growth and reproduction of *Thrips palmi* (Thysanoptera: Thripidae). Environ Entomol 24: 1598–1603.

312. Murai T (2000) Effect of temperature on development and reproduction of the onion thrips, *Thrips tabaci* Lindeman (Thysanoptera: Thripidae), on pollen and honey solution. Appl Entomol Zool 35: 499–504.
