# Supplementary material for: Thermodynamics Constrains Allometric Scaling of Optimal Development Time in Insects
Source: PLoS One. 2013 Dec 31;8(12):e84308. doi: 10.1371/journal.pone.0084308 (PMC3877264; doi:10.1371/journal.pone.0084308)
Supplement: Table S2 — Compiled equations for estimating dry mass. (DOC) [file pone.0084308.s004.doc]

**Table S2.**  Literature equations for dry mass (mg) as a function of body length (mm). Sample sizes are given in parentheses.

| Taxon (N) | Rogers et al. 1977^1^ | Smock 1980^2^ | Schoener 1980^3^ | Sample et al. 1993^4^ | Hodar 1996^5^ | Ganihar 1997^6^ | Benke 1999^7^ |
| --- | --- | --- | --- | --- | --- | --- | --- |
| Blattaria |  |  |  |  | 0.0494L^2.344^ (10) |  |  |
| Coleoptera | 0.0314L^2.79^ (151) | 0.153L^2.18^ (161) | 0.082L^1.99^ (47) | 0.0389L^2.492^ (330) | 0.0410L^2.640^ (156) | 0.0380L^2.4625^ (175) | 0.0077L^2.910^ (12) |
| Collembola |  |  |  |  | 0.0024L^3.676^ (8) | 0.1534L^2.3002^ (10) |  |
| Dermaptera |  |  |  |  | 0.0015L^3.497^ (10) | 0.6361e^0.2037L^ (8)^10^ |  |
| Diptera | 0.0371L^2.366^ (84) | 0.005L^2.43^ (136) | 0.022L^2.42^ (171) | 0.0414L^2.213^ (257) | 0.0312L^2.392^ (36) | 0.0324L^2.5943^ (20) | 0.0025L^2.692^ (25) |
| Ephemeroptera |  | 0.007L^2.88^ (459) |  |  |  |  | 0.0071L^2.832^ (34) |
| Hemiptera^8^ |  |  |  |  |  |  |  |
| Auchenorryncha + Sternorryncha | 0.0366L^2.696^ (59) |  | 0.024L^2.31^ (36) | 0.0594L^2.225^ (106) | 0.0548L^2.354^ (12)^11^ | 0.0408L^2.3487^ (40) |  |
| Heteroptera | 0.0499L^2.270^ (34) | 0.031L^2.40^ (114) | 0.006L^3.13^ (14) | 0.0084L^3.075^ (70) | 0.0341L^2.688^ (21) | 0.0205L^2.7642^ (43) | 0.0108L^2.734^ (4) |
| Hymenoptera^9^ | 0.0208L^2.407^ (97) |  | 0.016L^2.55^ (82) | 0.0138L^2.696^ (274) | 0.1636L^1.900^ (24) | 0.0276L^2.6429^ (26) |  |
| Lepidoptera | 0.0177L^2.903^ (28) |  | 0.014L^2.55^ (18) | 0.0065L^3.122^ (384) | 0.0095L^2.969^ (20) | 0.0083L^2.8585^ (10) |  |
| Neuroptera |  |  |  | 0.0113L^2.570^ (70) | 0.0814L^1.530^ (10) |  |  |
| Odonata |  | 0.014L^2.78^ (43) |  |  |  |  | 0.0078L^2.792^ (18) |
| Orthoptera | 0.0488L^2.515^ (35) |  |  |  | 0.0255L^2.637^ (27) | 0.0292L^2.4619^ (10) |  |
| Phasmatodea |  |  |  |  |  |  |  |
| Psocodea |  |  |  |  | 0.0425L^1.637^ (6) |  |  |
| Siphonaptera |  |  |  |  |  |  |  |
| Thysanoptera |  |  |  |  | 0.0071L^2.537^ (6) |  |  |
| All Insects | 0.0305L^2.62^ (500)^12^ |  | 0.024L^2.35^ (392) | 0.0266L^2.494^ (1673) | 0.0315L^2.492^ (448) | 0.0464L^2.2968^ (401) | 0.0064L^2.788^ (155) |

^1^ Shrub-steppe insects from Hanford, WA ^2^Aquatic insects from NC ^3^Forest insects from MA ^4^Forest insects from WV ^5^Insects from shrub-steppe, cereal crops, and fallow land in SE Spain ^6^Insects from near Goa, India ^7^Aquatic insects from AL, GA, ME, NC, SC, and VA. N's are number of species/equations included in the analysis. These data may not be completely independent from other data sets. ^8^Hemiptera was previously the order Heteroptera (“true bugs”). Heteroptera is now a suborder within Hemiptera. Auchenorrhyncha and Sternorrhyncha are suborders within Hemiptera that were previously lumped as the Homoptera. ^9^Excluding ants, family Formicidae ^10^An exponential rather than a power function was used. ^11^This excludes the family Aphididae which was calculated separately (0.0598L^1.724^, N=6) ^12^This general formula comes from the companion paper (Rogers et al., 1976). The actual N is not given: “Nearly 500 … ” All reference information is in the main text.
