# Supplementary material for: Thermodynamics Constrains Allometric Scaling of Optimal Development Time in Insects
Source: PLoS One. 2013 Dec 31;8(12):e84308. doi: 10.1371/journal.pone.0084308 (PMC3877264; doi:10.1371/journal.pone.0084308)
Supplement: Text S2 — Description of and references for phylogenetic tree construction. (DOC) [file pone.0084308.s006.doc]

## **Thermodynamics constrains allometric scaling of optimal development time in insects**

Dillon and Frazier

## **Text S2: Phylogenetic tree construction**

To build an hypothesis for relationships among the insect species included in our analyses, we used a heirarchical approach. We started with the broadest relationships (orders) and then drilled down to successively smaller and smaller groupings (family, subfamily, tribe, etc.) until we developed species relationships, relying on the tree of life [1,2] and Encyclopedia of Life to determine taxon classifications when necessary. We relied primarily on published molecular phylogenies, using the most recent and/or best resolved phylogeny wherever possible. In rare cases where multiple phylogenies revealed different relationships, we chose those relationships that were best supported (by multiple papers or higher confidence within a paper) or left the disputed relationship as a polytomy. Whenever there were only two species at a tip, we automatically coded them as sisters based on taxonomy (e.g. two species within an order or genus). Because phylogenetic relationships at the species level are largely unresolved for many groups, we were forced to code many polytomies, particularly at the species level. We started with Treebase Tr3310, which is Fig. 5 from [3], for the relationships among the major insect orders, which is in agreement with other phylogenetic hypotheses for the orders included in this study [4,5].

*Orders with fewer species*

The single Phasmatodia species was placed at that tip. For Blattaria and Odonata, the two representatives in each order were just coded as sisters. The 4 Collembola were based on [6], the 3 Dermaptera on [7], the 3 Ephemeroptera on [8], and the 3 Psocodea on [9]. The 6 Neuroptera were based on [10] and taxonomic relationships. The Siphonaptera relationships (6 species) were based on [11,12]. The relationships among the 10 species of Thysanoptera were based on [13], but the relationship between Megalurothrips and Scolothrips were unclear, so were based on taxonomy (<http://keys.lucidcentral.org/keys/v3/thrips_of_california/identify-thrips/key/california-thysanoptera-2012/Media/Html/browse_species/Megalurothrips_usitatus.htm>). The relationships among the 11 species of Orthoptera were based on [14–17].

*Coleoptera*

The relationships among beetle families is based on a comprehensive molecular phylogeny [18] which includes all of the families in our study and is in close agreement with other molecular phylogenetic [19–21] and morphological [22] hypotheses [19–22] The exception is the Derodontidae, which was historically considered part of the Bostrichiformia but which falls out as basal Polyphaga with strong support (Bayesian posterior probability of 97%) [18]. Many families (Anobiidae, Bostrichidae, Brentidae, Cerambycidae, Derodontidae, Melyridae, Nitudulidae, Scarabeidae) had 2 or fewer species that were simply placed at family tips. The 5 Carabidae, 3 Cucujidae, and 3 Sylvanidae species were coded as polytomies at their respective tips due to a lack of species-level data. Relationships among the 14 species in Chrysomelidae come primarily from [23], but *Leptinotarsa* was placed based on genus, *Gastrophysa* based on subfamily, and *Ceratoma* based on shared tribe (Diabroticina). Placement of *Callosobruchus* was based on subfamily, with species relationships based on [24]. Relationships among the 22 Coccinellidae species was based primarily on [25,26]. The only species not on the phylogeny was *Nephaspis oculatus*, which is supposed to be part of the Scymnini, so we placed it as sister to *Rhyzobius*, given the tree of [26]. Relationships among the 23 Curculionidae was based on [18,27,28], relying heavily on congeneric tree position. We could find very little data for the Dermestidae (8 species) or Tenebrionidae (13 species), but based them on taxonomic relationships, generic status, and [29] for *Tribolium* species.

*Diptera*

The overall Dipteran phylogeny and relationships among Brachycera families were from [30]. The following sources were used for relationships among taxonomic groups: Culicidae [31]; Drosophilidae, [32,33]; Chloropidae [34]; Agromyzidae [35], and within the genus *Liriomyza* (<http://www.fera.defra.gov.uk/plants/plantHealth/pestsDiseases/documents/protocols/liriomyza.pdf>, accessed on Dec 19, 2012); Calliphoridae [36]; Tephritidae [37], within the genus *Bactrocera* [38], and within the genus *Ceratitis* [39]; Muscidae [40–42], and within the genus *Musca* [43]; Tachinidae [44]. The placement of Cecidomyiidae and the genus *Atherigona* were problematic and based on taxonomy.

*Hemiptera*

The higher level phylogeny (placement of Sternorrhyncha, Auchenorrhyncha, and Heteroptera) are from [45], with higher levels of Sternorrhyncha from [46], of Auchenorrhyncha from [47], and of Heteroptera from [48,49]. Lower levels were resolved as follows: Psyllidae [50]; Pseudococcidae [51]; Aleyroididae [52]; Aphididae [53], with the Macrosiphini within Aphididae resolved following [54]; Gerromorpha [55]; Anthocoridae [56,57]; Miridae [48], with the genus *Lygus* [58]; Pentatomidae [59], with tribe Asopinae [60]. Placement of *Lipaphis*, and *Orius albidipennis* was unclear, so based loosely on taxonomy and phylogeography. The Pentatomidae were not well resolved in general with placement of many species based on subfamily membership and the placement of *Halyomorpha halys* was based on species groupings in the tribes Pentatomini and Cappaeini.

*Hymenoptera*

The family level tree was based on [61–63]. The placement of lower-level taxa was as follows: Braconidae [64], with the Aphidiinae [65] and Microgastrinae [66]; Platygastroidea [67]; Chalcidoidea [68] with Trichogrammatidae [69,70], Encrytidae and Aphelinidae [68]. Resolution of the Pteromalidae and of species relationships within most groups was poor.

*Lepidoptera*

Family relationships were based on [71–75], with some controversy surrounding the placement of basal groups Gelechioidea, Yponomeutoidea and Torticidae [75]. Most species relationships came from these large trees, except relationships within the Noctuidae [76].

**References**

1. Tree of Life Web Project (2002) Insecta (under construction). Tree of Life Web. Available:http://tolweb.org/Insecta/8205/2002.01.01 in The Tree of Life Web Project, http://tolweb.org/.

2. Wilson EO (2003) The encyclopedia of life. Trends in Ecology & Evolution 18: 77–80.

3. Whiting MF, Carpenter JC, Wheeler QD, Wheeler WC (1997) The Strepsiptera Problem: Phylogeny of the Holometabolous Insect Orders Inferred from 18S and 28S Ribosomal DNA Sequences and Morphology. Syst Biol 46: 1–68. doi:10.1093/sysbio/46.1.1.

4. Wheeler W (2001) The Phylogeny of the Extant Hexapod Orders. Cladistics 17: 113–169. doi:10.1006/clad.2000.0147.

5. Yeates DK, Cameron SL, Trautwein M (2012) A view from the edge of the forest: recent progress in understanding the relationships of the insect orders. Australian Journal of Entomology 51: 79–87. doi:10.1111/j.1440-6055.2012.00857.x.

6. Xiong Y, Gao Y, Yin W, Luan Y (2008) Molecular phylogeny of Collembola inferred from ribosomal RNA genes. Molecular phylogenetics and evolution 49: 728–735.

7. Jarvis KJ, Haas F, Whiting MF (2005) Phylogeny of earwigs (Insecta: Dermaptera) based on molecular and morphological evidence: reconsidering the classification of Dermaptera. Systematic Entomology 30: 442–453.

8. Ogden TH, Whiting MF (2005) Phylogeny of Ephemeroptera (mayflies) based on molecular evidence. Molecular Phylogenetics and Evolution 37: 625–643.

9. Yoshizawa K, Johnson KP (2010) How stable is the “Polyphyly of Lice” hypothesis (Insecta: Psocodea)?: A comparison of phylogenetic signal in multiple genes. Molecular Phylogenetics and Evolution 55: 939–951. doi:10.1016/j.ympev.2010.02.026.

10. Haruyama N, Mochizuki A, Duelli P, Naka H, Nomura M (2008) Green lacewing phylogeny, based on three nuclear genes (Chrysopidae, Neuroptera). Systematic Entomology 33: 275–288.

11. Whiting MF (2002) Mecoptera is paraphyletic: multiple genes and phylogeny of Mecoptera and Siphonaptera. Zoologica Scripta 31: 93–104.

12. Whiting MF, Whiting AS, Hastriter MW, Dittmar K (2008) A molecular phylogeny of fleas (Insecta: Siphonaptera): origins and host associations. Cladistics 24: 677–707. doi:10.1111/j.1096-0031.2008.00211.x.

13. Inoue T, Sakurai T (2007) The phylogeny of thrips (Thysanoptera: Thripidae) based on partial sequences of cytochrome oxidase I, 28S ribosomal DNA and elongation factor-1 α and the association with vector competence of tospoviruses. Applied Entomology and Zoology 42: 71–81. doi:10.1303/aez.2007.71.

14. Rowell CHF, Flook PK (1998) Phylogeny of the Caelifera and the Orthoptera as derived from ribosomal gene sequences. Journal of Orthoptera Research: 147–156.

15. Flook PK, Klee S, Rowell CHF (1999) Combined molecular phylogenetic analysis of the Orthoptera (Arthropoda, Insecta) and implications for their higher systematics. Systematic Biology 48: 233–253.

16. Huang Y (2000) Phylogenetic Relationships of North American Field Crickets Inferred from Mitochondrial DNA Data. Molecular Phylogenetics and Evolution 17: 48–57. doi:10.1006/mpev.2000.0815.

17. Jang Y, Gerhardt HC, Choe JC (2008) A comparative study of aggressiveness in eastern North American field cricket species (genus Gryllus). Behavioral Ecology and Sociobiology 62: 1397–1407. doi:10.1007/s00265-008-0568-6.

18. Hunt T, Bergsten J, Levkanicova Z, Papadopoulou A, John OS, et al. (2007) A Comprehensive Phylogeny of Beetles Reveals the Evolutionary Origins of a Superradiation. Science 318: 1913–1916. doi:10.1126/science.1146954.

19. Caterino MS, Shull VL, Hammond PM, Vogler AP (2002) Basal relationships of Coleoptera inferred from 18S rDNA sequences. Zoologica Scripta 31: 41–49.

20. Hunt T, Vogler AP (2008) A protocol for large-scale rRNA sequence analysis: Towards a detailed phylogeny of Coleoptera. Molecular Phylogenetics and Evolution 47: 289–301. doi:10.1016/j.ympev.2007.11.029.

21. Marvaldi AE, Duckett CN, Kjer KM, Gillespie JJ (2009) Structural alignment of 18S and 28S rDNA sequences provides insights into phylogeny of Phytophaga (Coleoptera: Curculionoidea and Chrysomeloidea). Zoologica Scripta 38: 63–77. doi:10.1111/j.1463-6409.2008.00360.x.

22. Grimaldi DA, Engel MS (2005) Evolution of the Insects. Cambridge Univ Pr. p. Available:http://books.google.com/books?hl=en&lr=&id=Ql6Jl6wKb88C&oi=fnd&pg=PR15&dq=evolution+of+the+insects&ots=q9zxA1AozB&sig=gsVLtzUJT5AvAdlNs3Kor9qC58s. Accessed 25 July 2012.

23. Gómez-Zurita J, Hunt T, Kopliku F, Vogler AP (2007) Recalibrated Tree of Leaf Beetles (Chrysomelidae) Indicates Independent Diversification of Angiosperms and Their Insect Herbivores. PLoS ONE 2: e360. doi:10.1371/journal.pone.0000360.

24. Tuda M, Rönn J, Buranapanichpan S, Wasano N, Arnqvist G (2006) Evolutionary diversification of the bean beetle genus Callosobruchus (Coleoptera: Bruchidae): traits associated with stored-product pest status. Molecular Ecology 15: 3541–3551. doi:10.1111/j.1365-294X.2006.03030.x.

25. Seago AE, Giorgi JA, Li J, Ślipiński A (2011) Phylogeny, classification and evolution of ladybird beetles (Coleoptera: Coccinellidae) based on simultaneous analysis of molecular and morphological data. Molecular Phylogenetics and Evolution 60: 137–151. doi:10.1016/j.ympev.2011.03.015.

26. Magro A, Lecompte E, Magné F, Hemptinne J-L, Crouau-Roy B (2010) Phylogeny of ladybirds (Coleoptera: Coccinellidae): Are the subfamilies monophyletic? Molecular Phylogenetics and Evolution 54: 833–848. doi:10.1016/j.ympev.2009.10.022.

27. Cognato AI, Sperling FAH (2000) Phylogeny of Ips DeGeer Species (Coleoptera: Scolytidae) Inferred from Mitochondrial Cytochrome Oxidase I DNA Sequence. Molecular Phylogenetics and Evolution 14: 445–460. doi:10.1006/mpev.1999.0705.

28. Farrell BD, Sequeira AS, O’Meara BC, Normark BB, Chung JH, et al. (2001) The Evolution of Agriculture in Beetles (curculionidae: Scolytinae and Platypodinae). Evolution 55: 2011–2027. doi:10.1111/j.0014-3820.2001.tb01318.x.

29. Angelini DR, Jockusch EL (2008) Relationships among pest flour beetles of the genus Tribolium (Tenebrionidae) inferred from multiple molecular markers. Molecular Phylogenetics and Evolution 46: 127–141. doi:10.1016/j.ympev.2007.08.017.

30. Wiegmann BM, Trautwein MD, Winkler IS, Barr NB, Kim JW, et al. (2011) Episodic radiations in the fly tree of life. Proceedings of the National Academy of Sciences 108: 5690–5695.

31. Shepard JJ, Andreadis TG, Vossbrinck CR (2006) Molecular phylogeny and evolutionary relationships among mosquitoes (Diptera: Culicidae) from the northeastern United States based on small subunit ribosomal DNA (18S rDNA) sequences. Journal of medical entomology 43: 443–454.

32. Pelandakis M, Solignac M (1993) Molecular phylogeny of Drosophila based on ribosomal RNA sequences. Journal of molecular evolution 37: 525–543.

33. Goto SG, Kitamura HW, Kimura MT (2000) Phylogenetic Relationships and Climatic Adaptations in the< i> Drosophila takahashii</i> and< i> montium</i> Species Subgroups. Molecular phylogenetics and evolution 15: 147–156.

34. Stiner FM, Johnson FM, Axtell RC (1971) Differentiation of *Hippelates pusio*, *H. bishoppi* and <i>H. pallipes<i> (Diptera: Chloropidae) by electrophoresis. Journal of Medical Entomology 8: 213–216.

35. Scheffer SJ, Winkler IS, Wiegmann BM (2007) Phylogenetic relationships within the leaf-mining flies (Diptera: Agromyzidae) inferred from sequence data from multiple genes. Molecular phylogenetics and evolution 42: 756–775.

36. Marinho MAT, Junqueira ACM, Paulo DF, Esposito MC, Villet MH, et al. (2012) Molecular phylogenetics of Oestroidea (Diptera: Calyptratae) with emphasis on Calliphoridae: insights into the inter-familial relationships and additional evidence for paraphyly among blowflies. Molecular Phylogenetics and Evolution. Available:http://www.sciencedirect.com/science/article/pii/S1055790312003168. Accessed 23 January 2013.

37. Han HY, Ro KE (2009) Molecular phylogeny of the family Tephritidae (Insecta: Diptera): new insight from combined analysis of the mitochondrial 12S, 16S, and COII genes. Molecules and cells 27: 55–66.

38. Zhang B, Liu YH, Wu WX, Wang ZL (2010) Molecular phylogeny of Bactrocera species (Diptera: Tephritidae: Dacini) inferred from mitochondrial sequences of 16S rDNA and COI sequences. Florida Entomologist 93: 369–377.

39. Barr NB, McPheron BA (2006) Molecular phylogenetics of the genus< i> Ceratitis</i>(Diptera: Tephritidae). Molecular phylogenetics and evolution 38: 216–230.

40. Carvalho CJB (1989) Classifica\ccão de Muscidae (Diptera): uma proposta através da análise cladístical. Revista brasileira de Zoologia 6: 627–648.

41. Kutty SN (2009) Building the tree of life: Reconstructing the evolution of a recent and megadiverse branch (Calyptrates: Diptera). Available:http://www.scholarbank.nus.edu.sg/handle/10635/15870. Accessed 23 January 2013.

42. Dsouli N, Delsuc F, Michaux J, De Stordeur E, Couloux A, et al. (2011) Phylogenetic analyses of mitochondrial and nuclear data in haematophagous flies support the paraphyly of the genus< i> Stomoxys</i>(Diptera: Muscidae). Infection, Genetics and Evolution 11: 663–670.

43. Nihei SS, De Carvalho CJB (2007) Phylogeny and classification of Muscini (Diptera, Muscidae). Zoological Journal of the Linnean Society 149: 493–532.

44. Stireman JO (2002) Phylogenetic relationships of tachinid flies in subfamily Exoristinae (Tachinidae: Diptera) based on 28S rDNA and elongation factor-1α. Systematic Entomology 27: 409–435.

45. Cryan JR, Urban JM (2012) Higher-level phylogeny of the insect order Hemiptera: is Auchenorrhyncha really paraphyletic? Systematic Entomology 37: 7–21. doi:10.1111/j.1365-3113.2011.00611.x.

46. Dohlen CD, Moran NA (1995) Molecular phylogeny of the Homoptera: a paraphyletic taxon. Journal of Molecular Evolution 41: 211–223.

47. Ren-Huai D, Xue-Xin C, Zi-Zhong L (2008) Phylogeny of Deltocephalinae (Hemiptera: Cicadellidae) from China based on partial 16S rDNA and 28S rDNA D2 sequences combined with morphological characters. Acta Entomologica Sinica 51: 1055–1064.

48. Tian Y, Zhu W, Li M, Xie Q, Bu W (2008) Influence of data conflict and molecular phylogeny of major clades in Cimicomorphan true bugs (Insecta: Hemiptera: Heteroptera). Molecular phylogenetics and evolution 47: 581–597.

49. Park DS, Foottit R, Maw E, Hebert PDN (2011) Barcoding bugs: DNA-based identification of the true bugs (Insecta: Hemiptera: Heteroptera). PloS one 6: e18749.

50. Thao MLL, Clark MA, Burckhardt DH, Moran NA, Baumann P (2001) Phylogenetic analysis of vertically transmitted psyllid endosymbionts (Candidatus Carsonella ruddii) based on atpAGD and rpoC: comparisons with 16S–23S rDNA-derived phylogeny. Current microbiology 42: 419–421.

51. Downie DA, Gullan PJ (2004) Phylogenetic analysis of mealybugs (Hemiptera: Coccoidea: Pseudococcidae) based on DNA sequences from three nuclear genes, and a review of the higher classification. Systematic Entomology 29: 238–260.

52. Thao MLL, Baumann P (2004) Evolutionary relationships of primary prokaryotic endosymbionts of whiteflies and their hosts. Applied and environmental microbiology 70: 3401–3406.

53. Ortiz-Rivas B, Martínez-Torres D (2010) Combination of molecular data support the existence of three main lineages in the phylogeny of aphids (Hemiptera: Aphididae) and the basal position of the subfamily Lachninae. Molecular Phylogenetics and Evolution 55: 305–317.

54. Von Dohlen CD, Rowe CA, Heie OE (2006) A test of morphological hypotheses for tribal and subtribal relationships of Aphidinae (Insecta: Hemiptera: Aphididae) using DNA sequences. Molecular phylogenetics and evolution 38: 316–329.

55. McLennan DA, Brooks DR, McPhail JD (1988) The benefits of communication between comparative ethology and phylogenetic systematics: a case study using gasterosteid fishes. Canadian Journal of Zoology 66: 2177–2190.

56. Jung S, Kim H, Yamada K, Lee S (2010) Molecular phylogeny and evolutionary habitat transition of the flower bugs (Heteroptera: Anthocoridae). Molecular phylogenetics and Evolution 57: 1173–1183.

57. Jung S, Lee S (2011) Phylogenetic and systematic study of Korean Orius species (Heteroptera: Anthocoridae) on the basis of molecular and morphological data. Applied entomology and zoology 46: 153–164.

58. Zhou C, Kandemir I, Walsh DB, Zalom FG, Lavine LC (2012) Identification of Lygus hesperus by DNA Barcoding Reveals Insignificant Levels of Genetic Structure among Distant and Habitat Diverse Populations. PloS one 7: e34528.

59. Tembe SS, Gaikwad SS, Shouche YS, Ghate, H. V. (2009) Barcoding true bug species of India.

60. Gapud VP (1981) A Generic Revision of the Subfamily Asopinae: With Consideration of Its Phylogenetic Position in the Family Pentatomidae and Superfamily Pentatomoidea (Hemiptera-Heteroptera) University of Kansas, Entomology.

61. Heraty J, Ronquist F, Carpenter JM, Hawks D, Schulmeister S, et al. (2011) Evolution of the hymenopteran megaradiation. Molecular Phylogenetics and Evolution 60: 73–88. doi:10.1016/j.ympev.2011.04.003.

62. Peters RS, Meyer B, Krogmann L, Borner J, Meusemann K, et al. (2011) The taming of an impossible child: a standardized all-in approach to the phylogeny of Hymenoptera using public database sequences. BMC Biology 9: 55. doi:10.1186/1741-7007-9-55.

63. Sharkey MJ, Carpenter JM, Vilhelmsen L, Heraty J, Liljeblad J, et al. (2012) Phylogenetic relationships among superfamilies of Hymenoptera. Cladistics 28: 80–112. doi:10.1111/j.1096-0031.2011.00366.x.

64. Sharanowski BJ, Dowling APG, Sharkey MJ (2011) Molecular phylogenetics of Braconidae (Hymenoptera: Ichneumonoidea), based on multiple nuclear genes, and implications for classification. Systematic Entomology 36: 549–572. doi:10.1111/j.1365-3113.2011.00580.x.

65. Sanchis A, Latorre A, González-Candelas F, Michelena JM (2000) An 18S rDNA-Based Molecular Phylogeny of Aphidiinae (Hymenoptera: Braconidae). Molecular Phylogenetics and Evolution 14: 180–194. doi:10.1006/mpev.1999.0701.

66. Michel-Salzat A, Whitfield JB (2004) Preliminary evolutionary relationships within the parasitoid wasp genus Cotesia (Hymenoptera: Braconidae: Microgastrinae): combined analysis of four genes. Systematic Entomology 29: 371–382. doi:10.1111/j.0307-6970.2004.00246.x.

67. Murphy NP, Carey D, Castro LR, Dowton M, Austin AD (2007) Phylogeny of the platygastroid wasps (Hymenoptera) based on sequences from the 18S rRNA, 28S rRNA and cytochrome oxidase I genes: implications for the evolution of the ovipositor system and host relationships. Biological Journal of the Linnean Society 91: 653–669. doi:10.1111/j.1095-8312.2007.00825.x.

68. Munro JB, Heraty JM, Burks RA, Hawks D, Mottern J, et al. (2011) A Molecular Phylogeny of the Chalcidoidea (Hymenoptera). PLoS ONE 6: e27023. doi:10.1371/journal.pone.0027023.

69. Schilthuizen MO, Stouthamer R (1997) Horizontal transmission of parthenogenesis–inducing microbes in Trichogramma wasps. Proc R Soc Lond B 264: 361–366. doi:10.1098/rspb.1997.0052.

70. Owen AK, George J, Pinto JD, Heraty JM (2007) A molecular phylogeny of the Trichogrammatidae (Hymenoptera: Chalcidoidea), with an evaluation of the utility of their male genitalia for higher level classification. Systematic Entomology 32: 227–251. doi:10.1111/j.1365-3113.2006.00361.x.

71. Regier JC, Fang QQ, Mitter C, Peigler RS, Friedlander TP, et al. (1998) Evolution and phylogenetic utility of the period gene in Lepidoptera. Molecular Biology and Evolution 15: 1172–1182.

72. Regier JC, Brown JW, Mitter C, Baixeras J, Cho S, et al. (2012) A Molecular Phylogeny for the Leaf-Roller Moths (Lepidoptera: Tortricidae) and Its Implications for Classification and Life History Evolution. PLoS ONE 7: e35574. doi:10.1371/journal.pone.0035574.

73. Bucheli SR, Wenzel J (2005) Gelechioidea (Insecta: Lepidoptera) systematics: A reexamination using combined morphology and mitochondrial DNA data. Molecular phylogenetics and evolution 35: 380–394.

74. Tree of Life Web Project (2010) Ditrysia. Version 17 November 2010 (temporary). Available:http://tolweb.org/Ditrysia/11868 in The Tree of Life Web Project, http://tolweb.org/. Accessed 4 July 2012.

75. Cho S, Zwick A, Regier JC, Mitter C, Cummings MP, et al. (2011) Can Deliberately Incomplete Gene Sample Augmentation Improve a Phylogeny Estimate for the Advanced Moths and Butterflies (Hexapoda: Lepidoptera)? Systematic Biology 60: 782–796. doi:10.1093/sysbio/syr079.

76. Mitchell A, Mitter C, Regier JC (2005) Systematics and evolution of the cutworm moths (Lepidoptera: Noctuidae): evidence from two protein-coding nuclear genes. Systematic Entomology 31: 21–46. doi:10.1111/j.1365-3113.2005.00306.x.
